# Supplementary material for: Impact of military service on physical health later in life: a qualitative study of geriatric UK veterans and non-veterans
Source: BMJ Open. 2019 Jul 16;9(7):e028189. doi: 10.1136/bmjopen-2018-028189 (PMC6661607; doi:10.1136/bmjopen-2018-028189)
Supplement: Supplementary data [file bmjopen-2018-028189supp001.pdf]

Supplementary File 1

Topic guide for semi-structured interviews with veterans and non-veterans

Questions
Can you tell me about your employment history?
Probe: Which job(s) would you consider your "main" career?
Probe: How were the early years of your career? What was that experience like?
Probe: What were your main tasks?
Probe: Did you have a say in how you did your work?
Probe: How would you describe your work load?
Probe: What was the most challenging part of this role?
Probe: Were you ever physically injured at work (e.g. shot at, beaten up, threatened to be hurt, exposed to a toxic chemical/substance that could cause you serious harm, etc.)?
Probe: Were you ever involved in a life-threatening automobile accident or another kind of serious accident while at work?
Probe: Did any close colleagues of yours have a traumatic experience while on the job (e.g. assaulted, serious injured, threatened to be hurt)?
Probe: Did you ever do something (either accidentally or on purpose) that led to the serious injury or death of another person while on the job?
Probe: Sometimes people have experiences they don't want to talk about in interviews. Without telling me what it was, did you ever have a difficult or traumatic experience while at work that you haven't mentioned because you don't want to talk about it?
Probe: What were the highlights of this this role?
Probe: What did this role mean to you?
Probe: Why did you do this work?
Probe: What made you go into this type of work?
Are there any skills you feel you've come away with from your job(s)? Why or why not?
Probe: Were there any opportunities open to you as a result of this role that you wouldn't have experienced otherwise (e.g. travel, training, etc.)? Why or why not?
Probe: What has been the most important learning experience in your job? What did it teach you?
How would you describe your physical health over your lifetime?
Probe: How would you describe your current physical health?
Probe: Can you describe any key turning points in your health?

Probe: Have you received a diagnosis for any of your past/present physical health difficulties?

Probe: Do you think any factors had an impact on your physical health? Why or why not?

What impact did your job(s) have on your physical health?

Probe: Have you had any treatment or support for your physical health difficulties? Can you describe your experience of getting this treatment or support?

Generally speaking, how has this type of profession impacted the health of other people?

Can you describe your experience of leaving your profession (e.g. to retire, change roles, etc.)?

Probe: What was retiring/leaving this job like for you?

Probe: Did leaving your profession have an impact on your finances, social or family life?

Probe: How do you feel about your life now that you are retired/have left this job?

Probe: Was there any advice or support that would have been useful to you in making this change?

For all veteran participants:

Have you heard of the military Covenant?

Probe: How would you describe what a ‘veteran’ is?

Note: Participants were asked open-ended questions and subsequent probing questions depending on their response.
