# Supplementary material for: Impact of military service on physical health later in life: a qualitative study of geriatric UK veterans and non-veterans
Source: BMJ Open. 2019 Jul 16;9(7):e028189. doi: 10.1136/bmjopen-2018-028189 (PMC6661607; doi:10.1136/bmjopen-2018-028189)
Supplement: Author's manuscript [file bmjopen-2018-028189.draft_revisions.pdf]

BMJ Open is committed to open peer review. As part of this commitment we make the peer review history of every article we publish publicly available.

When an article is published we post the peer reviewers' comments and the authors' responses online. We also post the versions of the paper that were used during peer review. These are the versions that the peer review comments apply to.

The versions of the paper that follow are the versions that were submitted during the peer review process. They are not the versions of record or the final published versions. They should not be cited or distributed as the published version of this manuscript.

BMJ Open is an open access journal and the full, final, typeset and author-corrected version of record of the manuscript is available on our site with no access controls, subscription charges or pay-per-view fees (http://bmjopen.bmj.com).

If you have any questions on BMJ Open's open peer review process please email info.bmjopen@bmj.com

BMJ Open

The impact of military service on physical health later in life: A qualitative study of geriatric UK veterans and non-veterans.

Journal:	*BMJ Open*
Manuscript ID	bmjopen-2018-028189
Article Type:	Research
Date Submitted by the Author:	26-Nov-2018

Complete List of Authors:	Williamson, Victoria; King's College London,<br>Harwood, Hannah; King's College London<br>Greenberg, Karla; King's College London<br>Stevelink, Sharon; King's College London<br>Greenberg , N; King's College London

Keywords:	Veteran, Military, Physical health, Geriatric, Occupation

SCHOLARONE™ Manuscripts

The impact of military service on physical health later in life: A qualitative study of geriatric UK veterans and non-veterans.

Victoria Williamson[a*], Hannah Harwood[a], Karla Greenberg[a], Sharon Stevelink[a+], and Neil Greenberg[a+]

[+] Joint Last Authors

[a] King's Centre for Military Health Research, King's College London, Weston Education Centre, 10 Cutcombe Road, London, SE5 9RJ

***Correspondence:**

Dr Victoria Williamson

Address: Kings Centre for Military Health Research, King's College London, Weston Education Centre, 10 Cutcombe Road, London, SE5 9RJ, UK

Tel: +44 207 848 5347; Email: Victoria.williamson@kcl.ac.uk

Funding statement: This research was supported by a grant from the Royal British Legion's Aged Veteran's Fund, grant number AVF-TRBL03 (NG). This paper represents independent research part-funded by the National Institute for Health Research (NIHR) Biomedical Research Centre at South London and Maudsley NHS Foundation Trust and King's College London (SS). The views expressed are those of the authors and not necessarily those of the NHS, the NIHR or the Department of Health and Social Care.

For peer review only

Competing interests: All authors have completed the ICMJE uniform disclosure form at http://www.icmje.org/coi_disclosure.pdf and declare: authors had financial support from a Royal British Legion grant for the submitted work; no financial relationships with any organisations that might have an interest in the submitted work in the previous 3 years; no other relationships or activities that could appear to have influenced the submitted work.

Data sharing statement: No additional data are available.

Ethics approval: The National Health Service Research Ethics Committee (17/LO/0077).

For peer review only

Abstract

Objectives: Military veterans often experience physical health problems in later life; however, it remains unclear whether these problems are due to military service or are a feature of the ageing process. This study aimed to explore veteran and non-veteran perceptions of the impact of their occupation on their physical wellbeing later in life.

Design: Semi-structured qualitative interviews analysed using thematic analysis.

Setting: Interviews were conducted in face-to-face in participants' homes or via telephone.

Participants: 35 veterans (≥65 years); 25 non-veterans (≥65 years) were recruited, as well as a close companion of all participants for triangulation (n=60).

Results: Most veterans reported good physical health later in life which they attributed to the fitness they developed during military service. However, several veterans described challenges in maintaining their desired level of physically activity due to new commitments and limited sports facilities when they left service. Fewer non-veterans had experienced work-related fitness activities or exercise in their civilian jobs. Ongoing physical health difficulties, such as deafness, were perceived to be due to exposure to workplace hazards and appeared more common in veterans compared to non-veterans. Veterans also described greater reluctance than non-veterans to seek medical treatment for physical health difficulties, which could be challenging for close companions who had to provide informal care.

Conclusions: Military service was largely perceived to be beneficial for physical wellbeing; although when occupation-related physical health problems were experienced, many veterans were unwilling to seek treatment. These findings may inform clinicians of the needs of older veterans and highlight potential barriers to care.

For peer review only

Article summary

Strengths and limitations of this study:

- Largescale qualitative study (n=120) which recruited veterans and non-veterans aged over 65 years and a close companion of each participant.
- Semi-structured, qualitative interviews were conducted face-to-face in participant's homes or via telephone.
- Inclusion of the views of a close companion (spouse, child, close friend) to triangulate the study findings.

For peer review only

The UK Armed Forces (AF) veteran population is becoming increasingly elderly, with approximately 64% of UK AF veterans over the age of 65 [1,2]. Despite the sizeable and growing elderly UK AF veteran community, relatively little is understood about the impact of military service on the wellbeing of older UK AF veterans. Limited literature, mostly from the US, suggests there may be several health implications from military service [2–4]. However, as over 65s in the general population also commonly suffer from various physical health problems [5,6], whether health issues experienced by older veterans are related to UK military service or are a feature of ageing remains unclear.

The limited available evidence indicates that UK AF veterans may be more likely than the general population to experience physical health conditions that affect their daily functioning. For example, working age UK AF veterans commonly have hearing and musculoskeletal problems compared to non-veterans [2]. Nonetheless, military personnel are often fitter and healthier at the time of enlistment than the general population, leading to a phenomenon termed the "healthy warrior effect" [7]. This may explain why a 25% lower mortality risk for older Australian veterans compared with non-veterans has been observed [7]. Moreover, the high-levels of physical activity required in the AF as part of training, sport or deployment activities may offer some protective effects for health conditions later in life, with midlife exercise found to significantly reduce the risk of later high cholesterol and dementia (e.g. [8]). Finally, physical health problems can often have an impact on family members and caregivers. How health problems in working-age individuals can affect their families has been examined in previous studies [31,32], however comparatively little is known about the experiences of caregivers of older ex-military personnel.

A deeper understanding of the perceived long-term impact of AF service on physical health may inform clinical practice, identify whether there is a need to alter the way in which services for this population are provided, and highlight areas for occupational policy change.

For peer review only

We conducted in-depth, qualitative interviews with older veterans, older non-veterans, and a close companion (i.e. spouse, child, close friend) of each veteran/non-veteran participant. We aimed to explore perceptions of the impact of military vs non-military occupation on physical wellbeing later in life.

Method

Ethical approval for this study was granted by the NHS Camden & King's Cross Research Ethics Committee (17/LO/0077). All participants gave informed consent for their participation.

Study design

This was a qualitative study using in-depth interviews. The study was nested within a larger programme of research examining the impact of military service on physical, psychological and social functioning later in life.

Participants

Four sub-samples of participants were recruited: 1) 25 older veterans (≥65 years, five or more years of regular AF service) not known to have mental health problems; 2) 25 older non-veterans not known to have mental health problems (≥65 years, five or more years of work in a non-military occupation), 3) 10 older veterans with experiences of mental health issues (≥65 years, five or more years of AF service); and 4) a close companion of all participants (≥18 years, n=60). The 25 veterans without mental health diagnoses and 25 non-veterans were age (+/- 5 years) and gender matched. Exclusion criteria included: brain damage or cognitive impairment; inability to speak English; or current self-injurious behaviour/suicidal intent.

Assessments

Physical health. Veteran and non-veteran physical health and quality of life were measured using the 12-item Short Form Health Survey (SF-12)[9] and the EuroQol (EQ-5D)

For peer review only

[10]. Summary scores for the physical health component of the SF-12 were used (maximum possible score of 100), with high scores indicating better health state. The visual analogue scale (VAS) of EQ-5D was used which asks respondents to rate their overall health with 'worst imaginable health state' set at 0 and 'best imaginable health state' set at 100 [10]. The reliability and validity of both measures have been evidenced [9,10,33].These questionnaire assessments were completed by post (n=62) or online (n=58).

Qualitative interview schedule and procedure

The semi-structured interview questions focused on participants perceptions of their career, the impact of their career on their physical health, and their experiences of informal care for physical health problems (see Supplementary file 1). Close companions were asked for their perceptions of the veteran or non-veteran's career and its impact on physical health, as well as their own experiences of caring for the veteran/non-veteran.

The majority of interviews were conducted face-to-face (n=82), with 38 conducted via telephone. Interviews lasted for an average of 67 minutes (median = 65 minutes, SD = 0.02, IQR Range = 52:29 – 01:20:56 minutes). All interviews were digitally recorded and transcribed verbatim. Interviews were completed by 120 participants and thematic saturation was achieved.

Procedure

Purposive sampling was used. Veteran and non-veteran participants were initially identified by the clinical care team following attendance at GP surgeries, walk in centres, community groups or mental health services. The clinical care team sought permission for patient contact details to be shared with the research team. Given this agreement, participants were contacted by researchers with additional study information. Participants could also self-refer to the study via study advertisements. Companions were recruited by asking veteran/non-veterans if they had a spouse, child or close friend willing to participate with

For peer review only

them. Veteran/non-veterans provided their companion's contact details and companions were then contacted by the research team and sent a study information pack. A total of 86 veteran/non-veteran participants were approached to take part in the study, 60 of which were recruited (69.8% recruitment rate). Eligible individuals who did not participate were largely not contactable or had no close companion to take part. All participants were recruited between March and December 2017. All participants were given a £20 voucher as a thank you for their time.

Data analysis

Quantitative analysis. Data were entered and analysed using the statistical analysis software package SPSS (version 24). Missing data on the EQ-5D VAS and SF-12 were excluded from the analysis (EQ-5D VAS data missing for 3 non-veterans and 1 veteran; SF-12 data missing for 5 non-veterans and 6 veterans). T-tests, Fishers exact tests, and Kruskal-Wallis tests were used to determine whether statistically significant differences between groups existed, with p values <0.05 used to indicate statistical significance.

Qualitative analysis. All transcripts were entered into NVivo 11 (QSR International). Data were analysed using thematic analysis [11]. Data collection and analysis took place simultaneously to allow emerging topics of interest to be investigated further in later interviews and to determine whether thematic saturation had been reached [12]. A reflexive journal was kept throughout data collection and analysis by the primary researcher (VW) to recognise the influence of the researcher's prior experiences, thoughts and assumptions and avoid premature or biased interpretations of the data [13]. To ensure reliability, all codes and themes were independently reviewed by authors VW and HH. Disagreements between authors were infrequent and were resolved following discussion and re-examination of the data. Peer debriefing was conducted, and feedback regarding data interpretation and analysis was sought from co-authors SS, KG, and NG.

For peer review only

Patient involvement

The study involved patients in shaping the study design and outcome measures as the study materials were piloted with veterans (n=3) and non-veterans (n=2). Patients were also involved in the study recruitment as recommendations for potential recruitment sites were sought from Patient Participation Group (PPG) meetings. The findings were disseminated to participants via a study newsletter.

Results

Descriptive information

Participant demographic information can be found in Table 1. Overall, the 35 veterans had between 5-40 years of AF service, with 34.3% serving in the Naval Services, 51.4% serving in the Army, and 14.3% in the Royal Air Force (RAF, see Table 1). Close companions (n=60) were largely spouses (n=46, 76.7%) or close friends (n=8, 13.3%) and had a mean age of 68.4 years (SD 10.6).

Insert Table 1 around here

Self-reported physical health problems (e.g. arthritis, diabetes, high blood pressure, hearing loss, etc.) were similar in veterans both with and without diagnoses of mental health problems. Veterans without diagnoses of mental health difficulties more often considered that their physical health conditions to AF service compared to veterans with mental health diagnoses. Differences in scores on the SF-12 and EQ-5D were not statistically significant between veteran, veterans with mental health diagnoses, and non-veteran samples (p>0.05).

Qualitative results

For peer review only

Three key themes emerged from the data regarding experiences and perceived impact of occupation-related exercise, exposure to workplace hazards and the effects on health, and perceptions of and responses to physical health problems. Anonymised participant comments are provided to illustrate the findings, and all participants have been given a pseudonym.

Occupation and physical exercise.

The majority of veterans reported high-levels of physical exercise in the AF, which was not experienced by non-veterans in civilian roles. Many veterans reported they had been very physically fit due to the military training exercises and sports. This high volume of exercise during their military career was seen as a key reason for veteran good physical health later in life. Many veterans continued to exercise once leaving the AF and keeping physically active was a central part of daily life in retirement. In roles where non-veterans had the opportunity to do some exercise (e.g. manual labour), this activity was considered to have kept them in good health at the time but was often seen as having no long-term benefits.

Veteran 1: I've always been pretty healthy, I used to do a lot of sport when I was in the Army…. [Now] I like to go for walks to keep me fit and I [have] a treadmill… So, mentally and physically I'm not too bad for my age.

Nonetheless, several veterans reported chronic arthritis and other musculoskeletal health complaints in older age thought to be due to the physically demanding activities required in the AF (e.g. parachuting, marching exercises, etc.). These health problems were less common in non-veterans and reportedly unrelated to occupation.

Veteran 2: It had an effect on my knees, osteoarthritis… When I joined the Army I did an awful lot of athletics… The Army doesn't do you any good. I mean you've only got

For peer review only

to come to a parade here and see the old RSM [Regimental Sergeant Major] banging his feet on the ground and all that sort of thing. And so, my knees are shot.

On leaving the AF, a minority of veterans experienced difficulties continuing physical activity, either due to the long working hours of their civilian job, being employed in a more sedentary civilian role (e.g. HGV driving, security, etc.), limited access to sports facilities, or due to a military-related physical injury. Veteran difficulties continuing exercise were also reported by their close companions.

Veteran 3: No, I haven't done any real sport since I went outside. There hasn't been the opportunity for it really…The [Sports] Centre here, I need two buses to get to it so… you know, puts you off a bit.

Impact of occupational health and safety.

Participants in both veteran and non-veteran samples reported sustaining physical injuries in the workplace, with the majority of injuries viewed as being due to a lack of protective equipment or poor health and safety regulations (e.g. lack of ear defenders, limited protection available against hazardous substances).

Non-veteran 2: We were made to do asbestos and they didn't even bother to give us a facemask. And the dangers weren't known in them days…One fellow was actually down the hatch and an [asbestos] flake landed on his nose and he licked it off with his tongue…he swallowed it. But of course, it was a killer.

Veterans more frequently reported long-term health difficulties due to a lack of protective equipment compared to non-veterans. Veterans often described experiencing

For peer review only

hearing loss due to a lack of ear defenders in loud engine rooms or on shooting ranges, as well as skin cancers caused by having no sun protection during training activities or deployment.

Veteran 4: I was on the ranges…we were firing this anti-tank gun. Well, in them days ear defenders were unheard of... I went deaf in the right ear… [I now hear] a sort of whistling noise.

For non-veterans, those in physically demanding jobs often reported strain injuries due to heavy lifting or spending several hours standing. Physically demanding jobs exacerbated existing physical health conditions (e.g. existing musculoskeletal problems made worse by heavy lifting). Some non-veterans in healthcare roles or jobs requiring foreign travel (e.g. Foreign Office, employment with an international company, etc.) also contracted serious illnesses (i.e. shingles, dysentery, etc.). Such injuries/illnesses often had long-lasting effects, with physical pain or disability experienced into retirement.

Non-veteran 3: I was on duty… and the nurse in charge…said 'I've heard you're pregnant, great! Don't come any closer, my little boy's got rubella!' And I didn't... I didn't even go into the room, and two weeks later, I had rubella.

Non-veteran Close Companion 1: Through his plodding around the streets and that…his feet are very bad…They're deformed… He's had waterworks problems, he's got arthritis in both of his shoulders which we think is due to him being out in all weathers policing… he'd be out overnight in all weathers and get drenched and we think that's not helped his arthritis.

For peer review only

Impact of occupational hazards on the family. It reportedly became more challenging for close companions to cope with veteran and non-veteran's occupation-related health problems later in life, particularly in cases of veteran/non-veteran hearing loss or debilitating illness. These close companions reported feeling lonely as well as frustrated that their plans to exercise or travel as a family in retirement were now not feasible.

Non-veteran Close Companion 2: When he retired, we didn't expect the existence we've got now... We thought we'd have weekends away and go for walks and we can't go for a walk with him because he can't walk very far… [we] very rarely go out for a meal or anything… Life just ticks by now!

The health of close companions or other family members could also reportedly be affected by veteran and non-veteran's exposure to workplace hazards. For example, some close companions reported being exposed to asbestos on veteran/non-veteran's work clothes. This indirect exposure was thought to cause serious illness, and family members reportedly experienced cancer, infertility, breathing problems and other conditions as a result. When family members became unwell in such circumstances, veterans and non-veterans reported significant guilt and anxiety for their family members' wellbeing, often taking considerable steps to address the perceived harm they had caused (e.g. setting up a charity to help others with similar health problems, becoming their full-time carer, etc.).

Veteran 6: You just accepted that you'd had a big dose of radiation. That was health and safety in those days… There could have been some gene modification... [because my wife] had three miscarriages in the next year.

Perceptions of and responses to health problems.

Reluctance to seek medical treatment for physical health problems both during and following military service was commonly reported in veterans. This reluctance was largely not found in non-veterans. Unwillingness to admit physical health problems and access treatment was reportedly due to a sense of self-sufficiency instilled by the AF and a need to feel and be seen as 'tough.'

Veteran 8: It's a man thing! Compounded by the Army. You can't be seen to be a wimp! You are a wimp, but you can't be seen... You've got to keep a stiff upper lip… if you go to anything like an injection, there's no way you flinch! It might hurt, but you don't flinch! No!

Experiences of care and support. Close companions played a central role in encouraging veterans to access medical treatment, often accompanying them to appointments and ensuring medication was taken. In cases of serious illness/disability, many close companions of veterans described that veterans were extremely reluctant to accept informal care, such as assistance with getting dressed, daily hygiene, etc., often due to embarrassment or pride, and unwillingness to accept care was less common in non-veterans.

Veteran Close Companion 3: [The doctors] found this cancer on him. And then he said 'I'm not having any treatment'… I said 'Look, there's more than you here…You've got [your] grandson…and all [your] friends… You've just got to be a bit considerate'. Anyhow, he had [the treatment].

For peer review only

Providing support and care to unwilling veterans could be a frustrating experience for close companions who often felt their efforts were not appreciated. To cope, close companions positively reframed their experience, for example describing that the veteran would provide them with the same care if needed or seeking support from other family members and friends. Close companions of non-veterans faced less resistance to their provision of support.

Veteran Close Companion 4: He did not enjoy having things done for him, where he couldn't reach his feet or his back to wash properly…So I had to do that for him and he didn't like that. Got very, very upset over that…He thought I was trying to take control of his life. Which I wasn't!... [And] we would have an argument.

Discussion

The aim of this study was to explore the perceived impact of military and non-military occupations on physical health later in life. Our findings illustrate the perceived considerable benefits of occupation on wellbeing, particularly for those who served in the AF, such as long-term good physical health as a result of physical exercise. At the same time, these results also highlight the significant long-term implications of workplace practices on wellbeing, including chronic physical health problems due to a lack of protective equipment and beliefs regarding the wider impact of such workplace hazards on an employee's family.

Our findings delineate the positive influences of occupation on health, including the perceived long-lasting benefits of physical exercise in the AF. Physical exercise is associated with lower risk of health problems later in life, including dementia [8] and cardiovascular issues [24]. This may potentially mean AF veterans are at an advantage as opportunities for occupational physical activity were more limited in non-veterans and perceived long-lasting health benefits of occupational exertion in this group were not described. However, as we

For peer review only

found no significant differences in physical functioning between veterans and non-veterans on the SF-12 and EQ-5D VAS, this finding must interpreted cautiously. Moreover, some difficulties continuing physical exercise on leaving the AF were experienced due to logistical barriers or musculoskeletal injuries. This is in line with previous research in working age US and UK veterans [25–27] and provides insight into why some veterans may be vulnerable to obesity on leaving the AF [28,29]. Interventions to increase physical activity in non-veteran workplaces and additional support for veterans to facilitate exercise on leaving the AF may be beneficial. Nonetheless, it is important to stress that this exercise must be well managed, as veterans also reported experiencing musculoskeletal health complaints which they believed were caused by very strenuous AF physical activity.

Previous research into the impact of military service on physical health has also highlighted that, compared to the general population, working-age veterans are more likely to experience hearing difficulties, musculoskeletal problems and arthritis on leaving service [2,14,15]. The findings of this study provide support for these results, offer insight into why such physical health problems potentially occur, for example that hearing loss occurred due to lack of ear defenders, and what the perceived long-term implications can be. As ongoing health problems linked to lack of protective equipment were found in both veteran and non-veteran groups, this may reflect occupational health and safety standards of the time (e.g. 1950-1990's; [16,17]). In veterans, it could also reflect the inability of the Ministry of Defence (MoD) to apply health and safety legislation in an unpredictable conflict zone [18]. Given the adverse impact of workplace hazards, this highlights the importance of effective health and safety procedures (e.g. regular safety inspections, employee training, etc.) and the continued need for the employers to ensure appropriate measures are in place to protect personnel. Participants believed that their family members could also be indirectly exposed to workplace hazards which were thought to contribute to physical health problems (e.g.

For peer review only

respiratory difficulties, cancer, etc.). While ascertaining whether family members' health problems were a result of veteran/non-veteran occupational practices or other factors, such as attribution bias, was beyond the scope of this study, these findings are not inconsistent with previous research (e.g. [19]), and suggest a need for continued research regarding the long-term health implications of hazardous work environments. These results also suggest that employers should provide employees family members with accurate information about potential health threats in order to safeguard against potential ill-effects, allay fears and inform them when and how to access medical support.

Another key theme emerged in relation to treatment seeking as veterans appeared to be more reluctant than non-veterans to seek medical treatment for physical health problems or accept informal care - reportedly due to embarrassment or an AF instilled need to be 'tough.' As delays to necessary medical care can adversely impact wellbeing [20], this behaviour is concerning. As efforts have been successfully made to encourage help-seeking in those personnel/veterans experiencing mental health difficulties (e.g. [21]), a similar approach for promoting treatment seeking for physical health issues may also be beneficial. Furthermore, close companions often provided support for veterans to access medical treatment as well as informal care. In line with previous research in carers, refusal of care could be a challenging and distressing experience for some [22,23]. Older caregivers who provide care to a reluctant spouse have been found to report long-term marital unhappiness, feelings of entrapment in the role of carer, and distress from witnessing the deterioration in their spouse's condition [30]. Therefore, the families of older veterans may benefit from additional support and guidance related to their role as a caregiver. Further research is needed to ensure the provision of such support is accessible and appropriate.

The current study provided a perspective on an important, yet understudied, group in examining the impact of military service on wellbeing in older UK AF veterans. We recruited

For peer review only

a diverse sample of veterans and non-veterans from multiple professions and took steps to ensure the data was robust by triangulation with close companions. At the same time, there are limitations that must be considered. First, this study largely included male veterans/non-veterans, although this is broadly consistent with the gender distribution of the UK AF. Second, this study included only those veterans and non-veterans who agreed to contact by the research team or chose to self-refer in response to study advertisements. This may mean that participants had particularly salient occupation-related issues they wished to discuss. Furthermore, given the qualitative nature of the study, the findings reflect the lived experiences of a sample of veterans, non-veterans, and close companions and large-scale quantitative investigations would be useful in determining the generalizability of the results.

Despite these limitations, this study adds to the very limited research into the long-term physical health implications of military service on older UK veterans in several ways. First, by directly comparing the experiences of veterans and non-veterans, our results suggest that military service may have considerable beneficial effects on physical health later in life due to greater opportunities for exercise. Second, these findings demonstrate that health and safety practices, particularly in the AF, in may lead to physical health problems which can impact long-term functioning. Finally, this research highlights that additional advice and support may be beneficial to veterans and their families to facilitate veteran access to medical treatment which could ultimately improve their wellbeing and reduce familial stress.

For peer review only

Contributors: VW, SAMS, HH, KG, NG made substantial contributions to the conception of the study and analysis and interpretation of data for the study; drafted the article and revised it critically for important intellectual content; gave final approval of the version to be published and agreed to be accountable for all aspects of the article in ensuring that questions related to the accuracy or integrity of any part of the article are appropriately investigated and resolved. VW and HH contributed to acquisition of the data. Authors would like to thank Solent NHS Trust and its PPG for the guidance and support they provided throughout this project.

For peer review only

References

1 NEL Commissioning Support Unit. Developing mental health services for veterans in England engagement report. Oxford: 2016.

2 The Royal British Legion. A UK Household Survey of the Ex-Service Community. London: 2014.

3 Lehavot K, Hoerster KD, Nelson KM, *et al.* Health Indicators for Military, Veteran, and Civilian Women. *American Journal of Preventive Medicine* 2012;**42**:473–80. doi:10.1016/j.amepre.2012.01.006

4 Bedard K, Deschênes O. The Long-Term Impact of Military Service on Health: Evidence from World War II and Korean War Veterans. *American Economic Review* 2006;**96**:176–94. doi:10.1257/000282806776157731

5 Blyth FM, Noguchi N. Chronic musculoskeletal pain and its impact on older people. *Best Practice & Research Clinical Rheumatology* 2017;**31**:160–8. doi:10.1016/j.berh.2017.10.004

6 Parker L, Moran GM, Roberts LM, *et al.* The burden of common chronic disease on health-related quality of life in an elderly community-dwelling population in the UK. *Family Practice* 2014;**31**:557–63. doi:10.1093/fampra/cmu035

7 McLaughlin R, Nielsen L, Waller M. An Evaluation of the Effect of Military Service on Mortality: Quantifying the Healthy Soldier Effect. *Annals of Epidemiology* Published Online First: 2008. doi:10.1016/j.annepidem.2008.09.002

8 Ahlskog JE, Geda YE, Graff-Radford NR, *et al.* Physical Exercise as a Preventive or Disease-Modifying Treatment of Dementia and Brain Aging. *Mayo Clinic Proceedings* 2011;**86**:876–84. doi:10.4065/mcp.2011.0252

9 Ware JE, Kosinski M, Keller SD. A 12 item short form health survey: Construction of scales and preliminary tests of reliability and validity. *Medical Care* Published Online First: 1996. doi:10.2307/3766749

10 Hurst NP, Jobanputra P, Hunter M, *et al.* Validity of EURO-QOL—a generic health status instrument—in patients with rheumatoid arthritis economic and health outcomes research group. *British Journal of Rheumatology* 1994.

11 Braun V, Clarke V. Using thematic analysis in psychology. *Qualitative Research in Psychology* 2006;**3**:77–101. doi:10.1191/1478088706qp063oa

12 Hennink MM, Kaiser BN, Marconi VC. Code Saturation Versus Meaning Saturation: How Many Interviews Are Enough? *Qualitative Health Research* 2017;**27**:591–608. doi:10.1177/1049732316665344

13 Morse JM. Critical Analysis of Strategies for Determining Rigor in Qualitative Inquiry. *Qualitative Health Research* 2015;**25**:1212–22. doi:10.1177/1049732315588501

For peer review only

14 Okpala NCE. Knowledge and Attitude of Infantry Soldiers to Hearing Conservation. *Military Medicine* 2007;**172**:520–2. doi:10.7205/MILMED.172.5.520

15 Williamson V, Diehle J, Dunn R, *et al.* The impact of military service on health and well-being. *Occupational Medicine* 2018. doi:10.1093/occmed/kqy139

16 Spurgeon A, Harrington JM, Cooper CL. Health and safety problems associated with long working hours: a review of the current position. *Occup Environ Med* 1997;**54**:367. doi:10.1136/oem.54.6.367

17 Mearns KJ, Flin R. Assessing the state of organizational safety—culture or climate? *Current Psychology* 1999;**18**:5–17. doi:10.1007/s12144-999-1013-3

18 SHE Health and Safety Software. Time for the MOD to lose its Crown Immunity? https://blog.shesoftware.com/time-mod-lose-crown-immunity (accessed 11 Jun 2018).

19 Rake C, Gilham C, Hatch J, *et al.* Occupational, domestic and environmental mesothelioma risks in the British population: a case–control study. *British Journal Of Cancer* 2009;**100**:1175.

20 Prentice JC, Pizer SD. Delayed Access to Health Care and Mortality. *Health Services Research* 2007;**42**:644–62. doi:10.1111/j.1475-6773.2006.00626.x

21 Clark-Hitt R, Smith SW, Broderick JS. Help a Buddy Take a Knee: Creating Persuasive Messages for Military Service Members to Encourage Others to Seek Mental Health Help. *Health Communication* 2012;**27**:429–38. doi:10.1080/10410236.2011.606525

22 Hirst M. Carer distress: A prospective, population-based study. *Social Science & Medicine* 2005;**61**:697–708. doi:10.1016/j.socscimed.2005.01.001

23 Aadil J, Shah O, Wadoo J, *et al.* Psychological Distress in Carers of People with Mental Disorders. *British Journal of Medical Practitioners* 2010;**33**.

24 Lavie Carl J., Arena Ross, Swift Damon L., *et al.* Exercise and the Cardiovascular System. *Circulation Research* 2015;**117**:207–19. doi:10.1161/CIRCRESAHA.117.305205

25 Almond N, Kahwati L, Kinsinger L, *et al.* The Prevalence of Overweight and Obesity among U.S. Military Veterans. *Military Medicine* 2008;**173**:544–9. doi:10.7205/MILMED.173.6.544

26 Ashwick R, Murphy D. Exploring the health risks of help-seeking military veterans living in different parts of the UK. *J R Army Med Corps* 2018;**164**:8. doi:10.1136/jramc-2017-000822

27 Johnelle Sparks P, Bollinger M. A Demographic Profile of Obesity in the Adult and Veteran US Populations in 2008. *Popul Res Policy Rev* 2011;**30**:211–33. doi:10.1007/s11113-010-9186-x

28 Ashby M. *How Does Military Experience Influence the Obesity Trend Among Retired Veterans: A Qualitative Grounded Theory Study*. Doctoral Dissertation. The University of the Rockies. 2017.

For peer review only

29 Tarlov E, Zenk SN, Matthews SA, *et al.* Neighborhood Resources to Support Healthy Diets and Physical Activity Among US Military Veterans. *Prev Chronic Dis* 2017;**14**. doi:10.5888/pcd14.160590

30 Murray J, Livingston G. A qualitative study of adjustment to caring for an older spouse with psychiatric illness. *Ageing & Society* 1998;**18**:659-71.

31 Thandi G, Oram S, Verey A, Greenberg N, Fear NT. Informal caregiving and intimate relationships: the experiences of spouses of UK military personnel. *Journal of the Royal Army Medical Corps* 2017;**163**:266-72.

32 Beach SR, Schulz R, Yee JL, Jackson S. Negative and positive health effects of caring for a disabled spouse: Longitudinal findings from the caregiver health effects study. *Psychology and Aging* 2000;**15**:259.

33 König HH, Heider D, Lehnert T, Riedel-Heller SG, Angermeyer MC, Matschinger H, Vilagut G, Bruffaerts R, Haro JM, de Girolamo G, de Graaf R. Health status of the advanced elderly in six European countries: results from a representative survey using EQ-5D and SF-12. *Health & Quality of Life Outcomes*. 2010;**8**:143.

For peer review only

Table 1

Participant demographic information

Demographic		Veterans with MH diagnosis n (%)	Veterans n (%)	Non-veterans n (%)	Close Companions n (%)
Age *M* (SD)		71.8 (6.5)	74.6 (6.9)	75.3 (7.5)	68.4 (10.6)
Gender	Male	7 (70.0)	22 (88.0)	22 (88.0)	51 (85.0)
	Female	3 (30.0)	3 (12.0)	3 (12.0)	9 (15.0)
Ethnicity	White British	10 (100.0)	25 (100.0)	24 (96.0)	59 (98.3)[a]
	Asian/Asian British	n/a	n/a	1 (4.0)	
Service Branch	Naval Services	5 (50.0)	7 (28.0)	n/a	n/a
	Army	4 (40.0)	14 (56.0)		
	RAF	1 (10.0)	4 (16.0)		
Service Length *M* (SD)		20.5 (12.8)	19.6 (8.4)	n/a	n/a
Non-veteran Professions	Doctor/nurse	n/a	n/a	3 (12.0)	n/a
	Managerial/office			6 (24.0)	
	Manual labour			5 (20.0)	

For peer review only

	Small business owner			3 (12.0)	
	Police			1 (4.0)	
	Engineer/scientist			4 (16.0)	
	Civil service			3 (12.0)	
CC Relationship	Spouse	7 (70.0)	16 (64.0)	23 (92.0)	n/a
	Child	1 (10.0)	3 (12.0)	1 (4.0)	
	Close friend	2 (20.0)	5 (20.0)	1 (4.0)	
	Niece/nephew	n/a	1 (4.0)	n/a	
Physical health score *M* (SD)		53.4 (2.4)	54.0 (2.9)	53.6 (2.3)	n/a
Quality of life *M* (SD)		66.4 (16.4)	77.2 (18.1)	70.7 (21.2)	n/a

Note. M= mean. SD= standard deviation. CC= close companion. CC Relationship = the close companion's relationship to the veteran or non-veteran. Veterans with MH diagnoses = veterans with a previous diagnosis of mental health disorders. Service length = number of years in military service. Physical health score = mean score on SF-12 physical health component. Quality of life = mean score on the EQ-5D VAS. Data missing for 3 non-veterans and one veteran (no mental health diagnosis) on the EQ-5D VAS. Data missing for 5 non-veterans and 6 veterans (no mental health diagnoses) on the SF-12. a= this demographic information was missing for one participant.

For peer review only

Supplementary File 1

Topic guide for semi-structured interviews with veterans and non-veterans

Questions
Can you tell me about your employment history?
Probe: Which job(s) would you consider your “main” career?
Probe: How were the early years of your career? What was that experience like?
Probe: What were your main tasks?
Probe: Did you have a say in how you did your work?
Probe: How would you describe your work load?
Probe: What was the most challenging part of this role?
Probe: Were you ever physically injured at work (e.g. shot at, beaten up, threatened to be hurt, exposed to a toxic chemical/substance that could cause you serious harm, etc.)?
Probe: Were you ever involved in a life-threatening automobile accident or another kind of serious accident while at work?
Probe: Did any close colleagues of yours have a traumatic experience while on the job (e.g. assaulted, serious injured, threatened to be hurt)?
Probe: Did you ever do something (either accidentally or on purpose) that led to the serious injury or death of another person while on the job?
Probe: Sometimes people have experiences they don’t want to talk about in interviews. Without telling me what it was, did you ever have a difficult or traumatic experience while at work that you haven’t mentioned because you don’t want to talk about it?
Probe: What were the highlights of this this role?
Probe: What did this role mean to you?

For peer review only

Probe: Why did you do this work?

Probe: What made you go into this type of work?

Are there any skills you feel you've come away with from your job(s)? Why or why not?

Probe: Were there any opportunities open to you as a result of this role that you wouldn't have experienced otherwise (e.g. travel, training, etc.)? Why or why not?

Probe: What has been the most important learning experience in your job? What did it teach you?

How would you describe your physical health over your lifetime?

Probe: How would you describe your current physical health?

Probe: Can you describe any key turning points in your health?

Probe: Have you received a diagnosis for any of your past/present physical health difficulties?

Probe: Do you think any factors had an impact on your physical health? Why or why not?

What impact did your job(s) have on your physical health?

Probe: Have you had any treatment or support for your physical health difficulties? Can you describe your experience of getting this treatment or support?

Generally speaking, how has this type of profession impacted the health of other people?

Can you describe your experience of leaving your profession (e.g. to retire, change roles, etc.)?

Probe: What was retiring/leaving this job like for you?

Probe: Did leaving your profession have an impact on your finances, social or family life?

Probe: How do you feel about your life now that you are retired/have left this job?

Probe: Was there any advice or support that would have been useful to you in making this change?

For peer review only

For all veteran participants:

Have you heard of the military Covenant?

Probe: How would you describe what a 'veteran' is?

Note: Participants were asked open-ended questions and subsequent probing questions depending on their response.

For peer review only

BMJ Open

The impact of military service on physical health later in life: A qualitative study of geriatric UK veterans and non-veterans.

Journal:	*BMJ Open*
Manuscript ID	bmjopen-2018-028189.R1
Article Type:	Research
Date Submitted by the Author:	29-Mar-2019
Complete List of Authors:	Williamson, Victoria; King's College London,<br>Harwood, Hannah; King's College London<br>Greenberg, Karla; King's College London<br>Stevelink, Sharon; King's College London<br>Greenberg , N; King's College London
**Primary Subject Heading**:	Occupational and environmental medicine
Secondary Subject Heading:	Geriatric medicine
Keywords:	Veteran, Military, Physical health, Geriatric, Occupation

SCHOLARONE™
Manuscripts

The impact of military service on physical health later in life: A qualitative study of geriatric UK veterans and non-veterans.

Victoria Williamson[a*], Hannah Harwood[a], Karla Greenberg[a], Sharon AM Stevelink[a,b+], and Neil Greenberg[a+]

[+] Joint Last Authors

[a] King's Centre for Military Health Research, King's College London, Weston Education Centre, 10 Cutcombe Road, London, SE5 9RJ

b Department of Psychological Medicine, King's College London, 16 DeCrespigny Park, London, postcode

***Correspondence:**

Dr Victoria Williamson

Address: Kings Centre for Military Health Research, King's College London, Weston Education Centre, 10 Cutcombe Road, London, SE5 9RJ, UK

Tel: +44 207 848 5347; Email: Victoria.williamson@kcl.ac.uk

Funding statement: This research was supported by a grant from the Royal British Legion's Aged Veteran's Fund, grant number AVF-TRBL03 (NG). This paper represents independent research part-funded by the National Institute for Health Research (NIHR) Biomedical Research Centre at South London and Maudsley NHS Foundation Trust and King's College

For peer review only - http://bmjopen.bmj.com/site/about/guidelines.xhtml

London (SS). The views expressed are those of the authors and not necessarily those of the NHS, the NIHR or the Department of Health and Social Care.

Competing interests: All authors have completed the ICMJE uniform disclosure form at http://www.icmje.org/coi_disclosure.pdf and declare: authors had financial support from a Royal British Legion grant for the submitted work; no financial relationships with any organisations that might have an interest in the submitted work in the previous 3 years; no other relationships or activities that could appear to have influenced the submitted work.

Data sharing statement: No additional data are available.

Ethics approval: The National Health Service Research Ethics Committee (17/LO/0077).

Acknowledgements: The authors would like to thank Solent NHS Trust and its PPG for the guidance and support they provided throughout this project.

For peer review only

Abstract

Objectives: Military veterans often experience physical health problems in later life; however, it remains unclear whether these problems are due to military service or are a feature of the ageing process. This study aimed to explore veteran and non-veteran perceptions of the impact of their occupation on their physical wellbeing later in life.

Design: Semi-structured qualitative interviews analysed using thematic analysis.

Setting: Interviews were conducted face-to-face in participants' homes or via telephone.

Participants: 35 veterans (≥65 years); 25 non-veterans (≥65 years) were recruited, as well as a close companion of all participants for triangulation (n=60).

Results: Most veterans reported good physical health later in life which they attributed to the fitness they developed during military service. However, several veterans described challenges in maintaining their desired level of physically activity due to new commitments and limited sports facilities when they left service. Fewer non-veterans had experienced work-related fitness activities or exercise in their civilian jobs. Ongoing physical health difficulties, such as deafness, were perceived to be due to exposure to workplace hazards and appeared more common in veterans compared to non-veterans. Veterans also described greater reluctance than non-veterans to seek medical treatment for physical health difficulties, which could be challenging for close companions who had to provide informal care.

Conclusions: Military service was largely perceived to be beneficial for physical wellbeing; although when occupation-related physical health problems were experienced, many veterans were unwilling to seek treatment. These findings may inform clinicians of the needs of older veterans and highlight potential barriers to care.

Keywords: Veteran, occupation, physical health, geriatric

For peer review only - http://bmjopen.bmj.com/site/about/guidelines.xhtml

Article summary

Strengths and limitations of this study:

- Large-scale qualitative study (n=120) which recruited veterans and non-veterans aged over 65 years and a close companion of each participant.
- Semi-structured, qualitative interviews were conducted face-to-face in participant's homes or via telephone.
- Inclusion of the views of a close companion (spouse, child, close friend) to triangulate the study findings.

For peer review only

The UK Armed Forces (AF) veteran population is becoming increasingly elderly, with approximately 64% of UK AF veterans over the age of 65 [1,2]. Despite the sizeable and growing elderly UK AF veteran community, relatively little is understood about the impact of military service on the wellbeing of older UK AF veterans. Limited literature, mostly from the US, suggests there may be several health implications from military service [2–4]. However, as over 65s in the general population also commonly suffer from various physical health problems [5,6], whether health issues experienced by older veterans are related to UK military service or are a feature of ageing remains unclear.

The limited available evidence indicates that UK AF veterans may be more likely than the general population to experience physical health conditions that affect their daily functioning. For example, working age UK AF veterans have higher rates of hearing and musculoskeletal problems compared to non-veterans [2]. Nonetheless, military personnel are often fitter and healthier at the time of enlistment than the general population, leading to a phenomenon termed the "healthy warrior effect" [7]. This may explain why a 25% lower mortality risk for older Australian veterans compared with non-veterans has been observed [7]. Moreover, the high-levels of physical activity required in the AF as part of training, sport or deployment activities may offer some protective effects for health conditions later in life, with midlife exercise found to significantly reduce the risk of later high cholesterol and dementia (e.g.[8]). Finally, physical health problems can often have an impact on family members and caregivers. How health problems in working-age individuals can affect their families has been examined in previous studies [9,10], however comparatively little is known about the experiences of caregivers of older ex-military personnel.

A deeper understanding of the perceived long-term impact of AF service on physical health may inform clinical practice, identify whether there is a need to alter the way in which services for this population are provided, and highlight areas for occupational policy change.

For peer review only - http://bmjopen.bmj.com/site/about/guidelines.xhtml

We conducted in-depth, qualitative interviews with older veterans, older non-veterans, and a close companion (i.e. spouse, child, close friend) of each veteran/non-veteran participant. We aimed to explore perceptions of the impact of military vs non-military occupation on physical wellbeing later in life.

Method

Ethical approval for this study was granted by the NHS Camden & King's Cross Research Ethics Committee (17/LO/0077). All participants gave informed consent for their participation.

Study design

This was a qualitative study using in-depth interviews. The qualitative approach utilised allowed for the exploration of issues that were most salient to participants and the subjective meaning attached to their career and wellbeing. The present study was nested within a larger programme of research [11] examining the impact of military service on physical, psychological and social functioning later in life.

Participants

Four sub-samples of participants were recruited: 1) 25 older veterans (≥65 years, five or more years of regular AF service) not known to have mental health problems; 2) 25 older non-veterans not known to have mental health problems (≥65 years, five or more years of work in a non-military occupation), 3) 10 older veterans with experiences of mental health issues (≥65 years, five or more years of AF service); and 4) a close companion of all participants (≥18 years, n=60). The 25 veterans without mental health diagnoses and 25 non-veterans were age (+/- 5 years) and gender matched. Exclusion criteria included: brain damage or cognitive impairment; inability to speak English; or current self-injurious behaviour/suicidal intent. This sample size was selected

Assessments

For peer review only

Physical health. Veteran and non-veteran physical health and quality of life were measured using the 12-item Short Form Health Survey (SF-12)[12] and the EuroQol (EQ-5D)[13]. Summary scores for the physical health component of the SF-12 were used (maximum possible score of 100), with high scores indicating better health state. The visual analogue scale (VAS) of EQ-5D was used which asks respondents to rate their overall health with 'worst imaginable health state' set at 0 and 'best imaginable health state' set at 100 [13]. The reliability and validity of both measures have been evidenced. These questionnaire assessments were completed by post (n=62) or online (n=58).

Qualitative interview schedule and procedure

The semi-structured interview questions focused on participants perceptions of their career, the impact of their career on their physical health, and their experiences of informal care for physical health problems (see Supplementary file 1). Close companions were asked for their perceptions of the veteran or non-veteran's career and its impact on physical health, as well as their own experiences of caring for the veteran/non-veteran.

The majority of interviews were conducted face-to-face (n=82), with 38 conducted via telephone. Interviews lasted for an average of 67 minutes (median = 65 minutes, SD = 0.02, IQR Range = 52:29 – 01:20:56 minutes). All interviews were digitally recorded and transcribed verbatim. Interviews were completed by 120 participants to represent a wide range in occupations and ages to reflect the possible diversity of opinions. Thematic saturation was achieved, and this was determined by the research team when no additional themes were found from the reviewing of successive data [14].

Procedure

Purposive sampling was used. As previous research has highlighted the relationship between poor mental health and physical health problems [2,15], the aim of the purposive sampling of veterans with self-reported mental health problems was to allow for further

For peer review only - http://bmjopen.bmj.com/site/about/guidelines.xhtml

exploration of potential links between occupation and health concerns. Veteran and non-veteran participants were initially identified by the clinical care team following attendance at National Health Service (NHS) GP surgeries, walk in centres, community groups or mental health services. The clinical care team identified potentially eligible participants using study inclusion/exclusion criteria. The clinical care team sought permission for patient contact details to be shared with the research team. Given this agreement, participants were contacted by researchers with additional study information. Participants could also self-refer to the study via study advertisements. Companions were recruited by asking veteran/non-veterans if they had a spouse, child or close friend willing to participate with them. Veteran/non-veterans provided their companion's contact details and companions were then contacted by the research team and sent a study information pack.

A total of 86 veteran/non-veteran participants were approached to take part in the study, 60 of which were recruited (69.8% recruitment rate). Eligible individuals who did not participate were largely not contactable or had no close companion to take part. All participants were recruited between March and December 2017. All participants were given a £20 voucher as a thank you for their time.

Data analysis

Quantitative analysis. Data were entered and analysed using the statistical analysis software package SPSS (version 24). Missing data on the EQ-5D VAS and SF-12 were excluded from the analysis (EQ-5D VAS data missing for 3 non-veterans and 1 veteran; SF-12 data missing for 5 non-veterans and 6 veterans). T-tests were used to determine whether statistically significant differences between groups existed, with p values <0.05 used to indicate statistical significance.

Qualitative analysis. All transcripts were entered into NVivo 11 (QSR International). Data were analysed using thematic analysis using the steps described by Braun and Clarke

For peer review only - http://bmjopen.bmj.com/site/about/guidelines.xhtml

[16]: reading and re-reading the data, producing codes, searching for and developing early themes, and revising and refining themes. An inductive analytical approach was used with initial codes and themes proposed by VW. Data collection and analysis took place simultaneously to allow emerging topics of interest to be investigated further in later interviews and to determine whether thematic saturation had been reached[14]. A reflexive journal was kept throughout data collection and analysis by the primary researcher (VW) to recognise the influence of the researcher's prior experiences, thoughts and assumptions and avoid premature or biased interpretations of the data[17]. To ensure reliability, all codes and themes were independently reviewed by authors VW and HH. Disagreements between authors were infrequent and were resolved following discussion and re-examination of the data. Peer debriefing was conducted to further enhance credibility, where discussions were held about the emerging findings and feedback regarding data interpretation and analysis was sought from co-authors SAMS, KG, and NG.

Patient involvement

The study involved patients in shaping the study design and outcome measures as the study materials were piloted with veterans (n=3) and non-veterans (n=2). Patients were also involved in the study recruitment as recommendations for potential recruitment sites were sought from Patient Participation Group (PPG) meetings. The findings were disseminated to participants via a study newsletter.

Results

Descriptive information

Participant demographic information can be found in Table 1. Overall, the 35 veterans had between 5-40 years of AF service, with 34.3% serving in the Naval Services, 51.4% serving in the Army, and 14.3% in the Royal Air Force (RAF, see Table 1). Close

For peer review only - http://bmjopen.bmj.com/site/about/guidelines.xhtml

companions (n=60) were largely spouses (n=46, 76.7%) or close friends (n=8, 13.3%) and had a mean age of 68.4 years (SD 10.6).

Insert Table 1 around here

Differences in scores on the SF-12 and EQ-5D were not statistically significant between veteran, veterans with mental health diagnoses, and non-veteran samples (p>0.05, see Table 1).

Qualitative results

Three key themes emerged from the data regarding experiences and perceived impact of occupation-related exercise, exposure to workplace hazards and the effects on health, and perceptions of and responses to physical health problems. Anonymised participant comments are provided to illustrate the findings in Table 2, and all participants have been given a pseudonym.

Occupation and physical exercise.

The majority of veterans reported high-levels of physical exercise in the AF, which was not described by non-veterans in civilian roles. Many veterans thought they had been very physically fit due to the military training exercises and sports. This high volume of exercise during their military career was seen as a key reason for veteran good physical health later in life. Many veterans continued to exercise once leaving the AF and keeping physically active was a central part of daily life in retirement. In roles where non-veterans had the opportunity to do some exercise (e.g. manual labour), this activity was considered to have kept them in good health at the time but was often seen as having no long-term benefits.

For peer review only - http://bmjopen.bmj.com/site/about/guidelines.xhtml

Nonetheless, several veterans stated they had chronic arthritis and other musculoskeletal health complaints in older age which were thought to be due to the physically demanding activities required in the AF (e.g. parachuting, marching exercises, etc.). These health problems were less commonly described by non-veterans and were not thought to be related to occupation.

On leaving the AF, a minority of veterans experienced difficulties continuing physical activity, either due to the long working hours of their civilian job, being employed in a more sedentary civilian role (e.g. HGV driving, security, etc.), limited access to sports facilities, or due to a military-related physical injury. Veteran difficulties continuing exercise were also discussed by their close companions.

Impact of occupational health and safety.

Participants in both veteran and non-veteran samples reported sustaining physical injuries in the workplace, with the majority of injuries viewed as being due to a lack of protective equipment or poor health and safety regulations (e.g. lack of ear defenders, limited protection available against hazardous substances).

Veterans more frequently reported long-term health difficulties due to a lack of protective equipment compared to non-veterans. Veterans often described experiencing hearing loss due to a lack of ear defenders in loud engine rooms or on shooting ranges, as well as skin cancers believed to be caused by having no sun protection during training activities or deployment.

For non-veterans, those in physically demanding jobs often reported strain injuries due to heavy lifting or spending several hours standing. Physically demanding jobs were thought to exacerbate existing physical health conditions (e.g. existing musculoskeletal problems made worse by heavy lifting). Some non-veterans in healthcare roles or jobs requiring foreign travel (e.g. Foreign Office, employment with an international company,

For peer review only

etc.) also described that they had contracted serious illnesses (i.e. shingles, dysentery, etc.). Such injuries/illnesses were often felt to have long-lasting effects, with physical pain or disability experienced into retirement.

Impact of occupational hazards on the family. It reportedly became more challenging for close companions to cope with veteran and non-veteran's occupation-related health problems later in life, particularly in cases of veteran/non-veteran hearing loss or debilitating illness. These close companions described feeling lonely as well as frustrated that their plans to exercise or travel as a family in retirement were now not feasible.

The health of close companions or other family members could also reportedly be affected by veteran and non-veteran's exposure to workplace hazards. For example, some close companions discussed being exposed to asbestos on veteran/non-veteran's work clothes. This indirect exposure was thought to cause serious illness, and family members reportedly experienced cancer, infertility, breathing problems and other conditions as a result. When family members became unwell in such circumstances, veterans and non-veterans disclosed experiencing significant guilt and anxiety for their family members' wellbeing, often taking considerable steps to address the perceived harm they had caused (e.g. setting up a charity to help others with similar health problems, becoming their full-time carer, etc.).

Perceptions of and responses to health problems.

Veterans with and without diagnoses of mental health problems described experiencing similar self-reported physical health problems (e.g. arthritis, diabetes, high blood pressure, hearing loss, etc.). Veterans without diagnoses of mental health difficulties more often considered that their physical health conditions related to AF service compared to veterans with mental health diagnoses. Few other differences in terms of physical health problems were described between the two sub-samples.

For peer review only - http://bmjopen.bmj.com/site/about/guidelines.xhtml

Reluctance to seek medical treatment for physical health problems both during and following military service was commonly reported in veterans. This reluctance was largely not described by non-veterans. Unwillingness to admit physical health problems and access treatment was reportedly due to a sense of self-sufficiency instilled by the AF and a need to feel and be seen as 'tough.'

Experiences of care and support. Close companions detailed how they play a central role in encouraging veterans to access medical treatment, often accompanying them to appointments and ensuring medication was taken. In cases of serious illness/disability, many close companions of veterans described that veterans were extremely reluctant to accept informal care, such as assistance with getting dressed, daily hygiene, etc., often due to embarrassment or pride, and unwillingness to accept care was less common in non-veterans.

Providing support and care to unwilling veterans could be a distressing and frustrating experience for close companions who often felt their efforts were not appreciated. To cope, close companions positively reframed their experience, for example describing that the veteran would provide them with the same care if needed or seeking support from other family members and friends. Close companions of non-veterans described facing less resistance to their provision of support.

Discussion

The aim of this study was to explore the perceived impact of military and non-military occupations on physical health later in life. Our findings illustrate the perceived considerable benefits of occupation on wellbeing, particularly for those who served in the AF, such as long-term good physical health as a result of physical exercise. At the same time, these results also highlight the significant long-term implications of workplace practices on wellbeing, including chronic physical health problems due to a lack of protective equipment and beliefs regarding the wider impact of such workplace hazards on an employee's family.

For peer review only - http://bmjopen.bmj.com/site/about/guidelines.xhtml

Our findings delineate the positive influences of occupation on health, including the perceived long-lasting benefits of physical exercise in the AF. Physical exercise is associated with lower risk of health problems later in life, including dementia [8] and cardiovascular issues [18]. This may potentially mean AF veterans are at an advantage as opportunities for occupational physical activity were more limited in non-veterans and perceived long-lasting health benefits of occupational exertion in this group were not described. However, as we found no significant differences in physical functioning between veterans and non-veterans on the SF-12 and EQ-5D VAS, this finding must interpreted cautiously. Moreover, some difficulties continuing physical exercise on leaving the AF were experienced due to logistical barriers or musculoskeletal injuries. This is in line with previous research in working age US and UK veterans [15,19,20] and provides insight into why some veterans may be vulnerable to obesity on leaving the AF [21,22]. Interventions to increase physical activity in non-veteran workplaces and additional support for veterans to facilitate exercise on leaving the AF may be beneficial. Nonetheless, it is important to stress that this exercise must be well managed, as veterans also reported experiencing musculoskeletal health complaints which they believed were caused by very strenuous AF physical activity.

Previous research into the impact of military service on physical health has also highlighted that, compared to the general population, working-age veterans are more likely to experience hearing difficulties, musculoskeletal problems and arthritis on leaving service [2,23,24]. The findings of this study provide support for these results, offer insight into why such physical health problems potentially occur, for example that hearing loss occurred due to lack of ear defenders, and what the perceived long-term implications can be. As ongoing health problems linked to lack of protective equipment were found in both veteran and non-veteran groups, this may reflect occupational health and safety standards of the time (e.g. 1950-1990's;[25,26]). In veterans, it could also reflect the inability of the Ministry of

For peer review only - http://bmjopen.bmj.com/site/about/guidelines.xhtml

Defence (MoD) to apply health and safety legislation in an unpredictable conflict zone[27]. Given the adverse impact of workplace hazards, this highlights the importance of effective health and safety procedures (e.g. regular safety inspections, employee training, etc.) and the continued need for the employers to ensure appropriate measures are in place to protect personnel. Participants believed that their family members could also be indirectly exposed to workplace hazards which were thought to contribute to physical health problems (e.g. respiratory difficulties, cancer, etc.). While ascertaining whether family members' health problems were a result of veteran/non-veteran occupational practices or other factors, such as attribution bias, was beyond the scope of this study, these findings are not inconsistent with previous research (e.g.[28]), and suggest a need for continued research regarding the long-term health implications of hazardous work environments. These results also suggest that employers should provide employees family members with accurate information about potential health threats in order to safeguard against potential ill-effects, allay fears and inform them when and how to access medical support.

Another key theme emerged in relation to treatment seeking as veterans appeared to be more reluctant than non-veterans to seek medical treatment for physical health problems or accept informal care - reportedly due to embarrassment or an AF instilled need to be 'tough.' As delays to necessary medical care can adversely impact wellbeing[29], this behaviour is concerning. As efforts have been successfully made to encourage help-seeking in those personnel/veterans experiencing mental health difficulties (e.g.[30]), a similar approach for promoting treatment seeking for physical health issues may also be beneficial. Furthermore, close companions often provided support for veterans to access medical treatment as well as informal care. In line with previous research in carers, refusal of care could be a challenging and distressing experience for some [31,32]. Older caregivers who provide care to a reluctant spouse have been found to report long-term marital unhappiness,

For peer review only - http://bmjopen.bmj.com/site/about/guidelines.xhtml

feelings of entrapment in the role of carer, and distress from witnessing the deterioration in their spouse's condition [33]. Therefore, the families of older veterans may benefit from additional support and guidance related to their role as a caregiver. Further research is needed to ensure the provision of such support is accessible and appropriate.

The current study provided a perspective on an important, yet understudied, group in examining the impact of military service on wellbeing in older UK AF veterans. We recruited a diverse sample of veterans and non-veterans from multiple professions and took steps to ensure the data was robust by triangulation with close companions. At the same time, there are limitations that must be considered. First, this study largely included male veterans/non-veterans, although this is broadly consistent with the gender distribution of the UK AF. Second, this study included only those veterans and non-veterans who agreed to contact by the research team or chose to self-refer in response to study advertisements. This may mean that participants had particularly salient occupation-related issues they wished to discuss. Third, while the key outcome measure of the study was the qualitative interview, a small proportion of participants did not fully complete questionnaire assessments resulting in missing data which must be taken into consideration. Furthermore, the inclusion of non-veterans with diagnoses of mental health problems in future studies would further our understanding of the potential relationship between occupation, physical and mental health. Finally, given the qualitative nature of the study, the findings reflect the lived experiences of a sample of veterans, non-veterans, and close companions and large-scale quantitative investigations would be useful in determining the generalizability of the results.

Despite these limitations, this study adds to the very limited research into the long-term physical health implications of military service on older UK veterans in several ways. First, by directly comparing the experiences of veterans and non-veterans, our results suggest that military service may have considerable beneficial effects on physical health later in life

For peer review only - http://bmjopen.bmj.com/site/about/guidelines.xhtml

due to greater opportunities for exercise. Second, these findings demonstrate that health and safety practices, particularly in the AF, in may lead to physical health problems which can impact long-term functioning. Finally, this research highlights that additional advice and support may be beneficial to veterans and their families to facilitate veteran access to medical treatment which could ultimately improve their wellbeing and reduce familial stress.

For peer review only

Contributors: VW, SAMS, HH, KG, NG made substantial contributions to the conception of the study and analysis and interpretation of data for the study; drafted the article and revised it critically for important intellectual content; gave final approval of the version to be published and agreed to be accountable for all aspects of the article in ensuring that questions related to the accuracy or integrity of any part of the article are appropriately investigated and resolved. VW and HH contributed to acquisition of the data.

For peer review only

For peer review only - http://bmjopen.bmj.com/site/about/guidelines.xhtml

1 NEL Commissioning Support Unit. Developing mental health services for veterans in England engagement report. 2016. https://www.england.nhs.uk/commissioning/wp-content/uploads/sites/12/2016/09/veterans-mh-services-engagement-rep.pdf (accessed 1 Aug 2018).

2 The Royal British Legion. A UK Household Survey of the Ex-Service Community. London: 2014. www.compasspartnership.co.uk

3 Lehavot K, Hoerster KD, Nelson KM, *et al.* Health Indicators for Military, Veteran, and Civilian Women. *Am J Prev Med* 2012;**42**:473–80. doi:10.1016/J.AMEPRE.2012.01.006

4 Bedard K, Deschênes O. The Long-Term Impact of Military Service on Health: Evidence from World War II and Korean War Veterans. *Am Econ Rev* 2006;**96**:176–94. doi:10.1257/000282806776157731

5 Blyth FM, Noguchi N. Chronic musculoskeletal pain and its impact on older people. *Best Pract Res Clin Rheumatol* 2017;**31**:160–8. doi:10.1016/J.BERH.2017.10.004

6 Parker L, Moran GM, Roberts LM, *et al.* The burden of common chronic disease on health-related quality of life in an elderly community-dwelling population in the UK. *Fam Pract* 2014;**31**:557–63. doi:10.1093/fampra/cmu035

7 McLaughlin R, Nielsen L, Waller M. An Evaluation of the Effect of Military Service on Mortality: Quantifying the Healthy Soldier Effect. *Ann Epidemiol* Published Online First: 2008. doi:10.1016/j.annepidem.2008.09.002

8 Ahlskog JE, Geda YE, Graff-Radford NR, *et al.* Physical Exercise as a Preventive or Disease-Modifying Treatment of Dementia and Brain Aging. *Mayo Clin Proc* 2011;**86**:876–84. doi:10.4065/MCP.2011.0252

9 Thandi G, Oram S, Verey A, *et al.* Informal caregiving and intimate relationships: the

For peer review only

For peer review only - http://bmjopen.bmj.com/site/about/guidelines.xhtml

experiences of spouses of UK military personnel. *J R Army Med Corps* 2017;**163**:266–72. doi:10.1136/jramc-2016-000679

10 Beach SR, Schulz R, Yee JL, *et al.* Negative and positive health effects of caring for a disabled spouse: Longitudinal findings from the Caregiver Health Effects Study. *Psychol Aging* 2000;**15**:259–71. doi:10.1037/0882-7974.15.2.259

11 Williamson, V., Harwood, H., Greenberg, K., Stevelink, S., Greenberg N. Veteran and Occupation Impact Evaluation (VOICE) Study. London: 2018. https://www.kcl.ac.uk/kcmhr/publications/reports/files/voice-study-report-2018.pdf (accessed 29 Mar 2019).

12 Ware JE, Kosinski M, Keller SD. A 12 item short form health survey: Construction of scales and preliminary tests of reliability and validity. *Med Care* Published Online First: 1996. doi:10.2307/3766749

13 Hurst NP, Jobanputra P, Hunter M, *et al.* Validity of EUROQOL—a generic health status instrument—in patients with rheumatoid arthritis economic and health outcomes research group. *Br J Rheumatol* 1994.

14 Hennink MM, Kaiser BN, Marconi VC. Code Saturation Versus Meaning Saturation. *Qual Health Res* 2017;**27**:591–608. doi:10.1177/1049732316665344

15 Ashwick R, Murphy D. Exploring the health risks of help-seeking military veterans living in different parts of the UK. *J R Army Med Corps* 2017;**0**:1–7. doi:10.1136/jramc-2017-000822

16 Braun V, Clarke V. Using thematic analysis in psychology. *Qual Res Psychol* 2006;**3**:77–101.

17 Morse JM. Critical Analysis of Strategies for Determining Rigor in Qualitative Inquiry. *Qual Health Res* 2015;**25**:1212–22. doi:10.1177/1049732315588501

18 Lavie CJ, Arena R, Swift DL, *et al.* Exercise and the Cardiovascular System. *Circ Res*

For peer review only - http://bmjopen.bmj.com/site/about/guidelines.xhtml

2015;**117**:207–19. doi:10.1161/CIRCRESAHA.117.305205

19 Almond N, Kahwati L, Kinsinger L, *et al.* Prevalence of overweight and obesity among U.S. military veterans. *Mil Med* 2008;**173**:544–9.http://www.ncbi.nlm.nih.gov/pubmed/18595417 (accessed 1 Aug 2018).

20 Johnelle Sparks P, Bollinger M. A Demographic Profile of Obesity in the Adult and Veteran US Populations in 2008. *Popul Res Policy Rev* 2011;**30**:211–33. doi:10.1007/s11113-010-9186-x

21 Ashby M. *How Does Military Experience Influence the Obesity Trend Among Retired Veterans: A Qualitative Grounded Theory Study*. Doctoral Dissertation. The University of the Rockies. 2017.

22 Tarlov E, Zenk SN, Matthews SA, *et al.* Neighborhood Resources to Support Healthy Diets and Physical Activity Among US Military Veterans. *Prev Chronic Dis* 2017;**14**:160590. doi:10.5888/pcd14.160590

23 Okpala NCE. Knowledge and Attitude of Infantry Soldiers to Hearing Conservation. *Mil Med* 2007;**172**:520–2. doi:10.7205/MILMED.172.5.520

24 Williamson V, Diehle J, Dunn R, *et al.* The impact of military service on health and well-being. *Occup Med (Chic Ill)* 2019;**69**:64–70. doi:10.1093/occmed/kqy139

25 Spurgeon A, Harrington JM, Cooper CL. Health and safety problems associated with long working hours: a review of the current position. *Occup Environ Med* 1997;**54**:367–75.http://www.ncbi.nlm.nih.gov/pubmed/9245942 (accessed 1 Aug 2018).

26 Mearns KJ, Flin R. Assessing the state of organizational safety—culture or climate? *Curr Psychol* 1999;**18**:5–17. doi:10.1007/s12144-999-1013-3

27 SHE Health & Safety Software. Time for the MOD to lose its Crown immunity? https://blog.shesoftware.com/time-mod-lose-crown-immunity (accessed 29 Mar 2019).

For peer review only - http://bmjopen.bmj.com/site/about/guidelines.xhtml

28 Rake C, Gilham C, Hatch J, *et al.* Occupational, domestic and environmental mesothelioma risks in the British population: a case–control study. *Br J Cancer* 2009;**100**:1175–83. doi:10.1038/sj.bjc.6604879

29 Prentice JC, Pizer SD. Delayed access to health care and mortality. *Health Serv Res* 2007;**42**:644–62. doi:10.1111/j.1475-6773.2006.00626.x

30 Clark-Hitt R, Smith SW, Broderick JS. Help a Buddy Take a Knee: Creating Persuasive Messages for Military Service Members to Encourage Others to Seek Mental Health Help. *Health Commun* 2012;**27**:429–38. doi:10.1080/10410236.2011.606525

31 Hirst M. Carer distress: A prospective, population-based study. *Soc Sci Med* 2005;**61**:697–708. doi:10.1016/J.SOCSCIMED.2005.01.001

32 Sha, A, Wadoo, O, Latoo J. Psychological distress in carers of people with mental disorders. *Br J Med Pract* 2010;**3**.www.bjmp.org (accessed 29 Mar 2019).

33 Murray, J, Livingston G. A qualitative study of adjustment to caring for an older spouse with psychiatric illness. *Ageing Soc* 1998;**18**:659–71.https://www.cambridge.org/core/journals/ageing-and-society/article/qualitative-study-of-adjustment-to-caring-for-an-older-spouse-with-psychiatric-illness/FD6CAB7E3B9ED18E875502A2220BFA3D (accessed 29 Mar 2019).

For peer review only

For peer review only - http://bmjopen.bmj.com/site/about/guidelines.xhtml

Table 1

Participant demographic information

Demographic		Veterans with MH diagnosis n (%), n=10	Veterans n (%), n=25	Non-veterans n (%), n=25	Close Companions n (%), n=60
Age *M* (SD)		71.8 (6.5)	74.6 (6.9)	75.3 (7.5)	68.4 (10.6)
Gender	Male	7 (70.0)	22 (88.0)	22 (88.0)	9 (15.0)
	Female	3 (30.0)	3 (12.0)	3 (12.0)	51 (85.0)
Ethnicity	White British	10 (100.0)	25 (100.0)	24 (96.0)	59 (98.0)[a]
	Asian/Asian British	n/a	n/a	1 (4.0)	
Service Branch	Naval Services	5 (50.0)	7 (28.0)	n/a	n/a
	Army	4 (40.0)	14 (56.0)		
	RAF	1 (10.0)	4 (16.0)		
Service Length *M* (SD)		20.5 (12.8)	19.6 (8.4)	n/a	n/a
Non-veteran Professions	Doctor/nurse	n/a	n/a	3 (12.0)	n/a
	Managerial/office			6 (24.0)	

For peer review only - http://bmjopen.bmj.com/site/about/guidelines.xhtml

	Manual labour			5 (20.0)	
	Small business owner			3 (12.0)	
	Police			1 (4.0)	
	Engineer/scientist			4 (16.0)	
	Civil service			3 (12.0)	
CC Relationship	Spouse	7 (70.0)	16 (64.0)	23 (92.0)	n/a
	Child	1 (10.0)	3 (12.0)	1 (4.0)	
	Close friend	2 (20.0)	5 (20.0)	1 (4.0)	
	Niece/nephew	n/a	1 (4.0)	n/a	
Physical health score *M* (SD)		53.4 (2.4)	54.0 (2.9)	53.6 (2.3)	n/a
Quality of life *M* (SD)		66.4 (16.4)	77.2 (18.1)	70.7 (21.2)	n/a

Note. M= mean. SD= standard deviation. CC= close companion. CC Relationship = the close companion's relationship to the veteran or non-veteran. Veterans with MH diagnoses = veterans with a previous diagnosis of mental health disorders. Service length = number of years in military service. Physical health score = mean score on SF-12 physical health component. Quality of life = mean score on the EQ-5D VAS. Data missing for 3 non-veterans and one veteran (no mental health diagnosis) on the EQ-5D VAS. Data missing for 5 non-veterans and 6 veterans (no mental health diagnoses) on the SF-12. a= this demographic information was missing for one participant. No significant differences between SF-12 scores were found between veterans and veterans with MH diagnoses (t(33)=0.96; p=0.35); veterans without mental disorders and non-veterans (t(48)=-1.20; p=0.24); and non-veterans and veterans with mental health diagnoses (t(33)=0.11; p=0.916). No significant differences

For peer review only - http://bmjopen.bmj.com/site/about/guidelines.xhtml

between EQ5D-VAS scores were found between veterans and veterans with MH diagnoses (t(32)=1.619; p=0.12); veterans without mental disorders and non-veterans (t(44)=-1.11; p=0.27); and non-veterans and veterans with mental health diagnoses (t(30)=0.57; p=0.57).

For peer review only

Table 2
Themes and sub-themes following thematic analysis

Themes and sub-themes	Findings	Verbatim quotes
Occupation and physical exercise		
High volume of exercise in AF	Veterans reported high-levels of physical exercise during their military career and this was considered a key reason for their good physical health later in life. Few opportunities for exercise were described in non-veterans in civilian roles.	Veteran 1: I've always been pretty healthy, I used to do a lot of sport when I was in the Army…. [Now] I like to go for walks to keep me fit and I [have] a treadmill… So, mentally and physically I'm not too bad for my age.
AF exercise and health problems	The high volume of exercise during military service was thought to have caused chronic health problems in some veterans later in life (e.g. arthritis). These problems were less common in non-veterans and reportedly unrelated to occupation.	Veteran 2: It had an effect on my knees, osteoarthritis… When I joined the Army I did an awful lot of athletics… The Army doesn't do you any good. I mean you've only got to come to a parade here and see the old RSM [Regimental Sergeant Major] banging his feet on the ground and all that sort of thing. And so, my knees are shot.
Difficulties continuing exercise on transition from the AF	Veterans described difficulties in continuing to exercise on transitioning from the military for several reasons, including a lack of sports facilities and more sedentary civilian roles.	Veteran 3: No, I haven't done any real sport since I went outside. There hasn't been the opportunity for it really…The [Sports] Centre here, I need two buses to get to it so... you know, puts you off a bit.
Impact of occupational health and safety		

For peer review only - http://bmjopen.bmj.com/site/about/guidelines.xhtml

Workplace injuries/illnesses	Both veterans and non-veterans described experiencing workplace injuries/illnesses which were often due to poor health and safety regulations. In particular, hearing loss was commonly described by veterans, whereas strain injuries were frequent discussed by non-veterans.	Non-veteran Close Companion 1: Through his plodding around the streets and that…his feet are very bad…They're deformed… He's had waterworks problems, he's got arthritis in both of his shoulders which we think is due to him being out in all weathers policing… he'd be out overnight in all weathers and get drenched and we think that's not helped his arthritis.
Impact of occupational hazards on the family	The health of family members was thought be affected by veteran and non-veteran's exposure to some workplace hazards. Indirect hazard exposure was believed to cause serious illness in family members which often caused veterans/non-veterans to feel guilty and concerned for their wellbeing.	Non-veteran 3: I was on duty… and the nurse in charge…said 'I've heard you're pregnant, great! Don't come any closer, my little boy's got rubella!' And I didn't... I didn't even go into the room, and two weeks later, I had rubella…and that's why my [child] was profoundly deaf.
Perceptions of and responses to health problems		
Reluctance to access care	Reluctance to seek medical treatment for physical health problems both during and following military service was common in veterans. This reluctance was largely not found in non-veterans. Unwillingness to access care was reportedly due to an AF instilled need to feel and be seen as 'tough.'	Veteran 8: It's a man thing! Compounded by the Army. You can't be seen to be a wimp! You are a wimp, but you can't be seen... You've got to keep a stiff upper lip… if you go to anything like an injection, there's no way you flinch! It might hurt, but you don't flinch! No!

For peer review only

For peer review only - http://bmjopen.bmj.com/site/about/guidelines.xhtml

Experiences of care and support	Veteran close companions described that ill/injured veterans were extremely reluctant to accept (in)formal care, often due to embarrassment or pride. Unwillingness to accept care was less common in non-veterans.	Veteran Close Companion 4: He did not enjoy having things done for him, where he couldn't reach his feet or his back to wash properly…So I had to do that for him and he didn't like that. Got very, very upset over that…He thought I was trying to take control of his life. Which I wasn't!... [And] we would have an argument.
Impact of providing care on the family	Providing care to unwilling veterans could be distressing and frustrating for close companions, who often felt their efforts were not appreciated. Close companions of non-veterans described facing less resistance to their provision of support.	Veteran Close Companion 3: [The doctors] found this cancer on him. And then he said 'I'm not having any treatment'… I said 'Look, there's more than you here…You've got [your] grandson…and all [your] friends… You've just got to be a bit considerate'. Anyhow, he had [the treatment].

Note: AF = Armed Forces. All participants have been assigned a pseudonym.

For peer review only - http://bmjopen.bmj.com/site/about/guidelines.xhtml

For peer review only

Supplementary File 1

Topic guide for semi-structured interviews with veterans and non-veterans

Questions
Can you tell me about your employment history?
Probe: Which job(s) would you consider your “main” career?
Probe: How were the early years of your career? What was that experience like?
Probe: What were your main tasks?
Probe: Did you have a say in how you did your work?
Probe: How would you describe your work load?
Probe: What was the most challenging part of this role?
Probe: Were you ever physically injured at work (e.g. shot at, beaten up, threatened to be hurt, exposed to a toxic chemical/substance that could cause you serious harm, etc.)?
Probe: Were you ever involved in a life-threatening automobile accident or another kind of serious accident while at work?
Probe: Did any close colleagues of yours have a traumatic experience while on the job (e.g. assaulted, serious injured, threatened to be hurt)?
Probe: Did you ever do something (either accidentally or on purpose) that led to the serious injury or death of another person while on the job?
Probe: Sometimes people have experiences they don’t want to talk about in interviews. Without telling me what it was, did you ever have a difficult or traumatic experience while at work that you haven’t mentioned because you don’t want to talk about it?
Probe: What were the highlights of this this role?
Probe: What did this role mean to you?
Probe: Why did you do this work?
Probe: What made you go into this type of work?
Are there any skills you feel you’ve come away with from your job(s)? Why or why not?
Probe: Were there any opportunities open to you as a result of this role that you wouldn’t have experienced otherwise (e.g. travel, training, etc.)? Why or why not?
Probe: What has been the most important learning experience in your job? What did it teach you?
How would you describe your physical health over your lifetime?
Probe: How would you describe your current physical health?
Probe: Can you describe any key turning points in your health?

For peer review only

Probe: Have you received a diagnosis for any of your past/present physical health difficulties?

Probe: Do you think any factors had an impact on your physical health? Why or why not?

What impact did your job(s) have on your physical health?

Probe: Have you had any treatment or support for your physical health difficulties? Can you describe your experience of getting this treatment or support?

Generally speaking, how has this type of profession impacted the health of other people?

Can you describe your experience of leaving your profession (e.g. to retire, change roles, etc.)?

Probe: What was retiring/leaving this job like for you?

Probe: Did leaving your profession have an impact on your finances, social or family life?

Probe: How do you feel about your life now that you are retired/have left this job?

Probe: Was there any advice or support that would have been useful to you in making this change?

For all veteran participants:

Have you heard of the military Covenant?

Probe: How would you describe what a 'veteran' is?

Note: Participants were asked open-ended questions and subsequent probing questions depending on their response.

For peer review only

BMJ Open

The impact of military service on physical health later in life: A qualitative study of geriatric UK veterans and non-veterans.

Journal:	*BMJ Open*
Manuscript ID	bmjopen-2018-028189.R2
Article Type:	Research
Date Submitted by the Author:	06-Jun-2019
Complete List of Authors:	Williamson, Victoria; King's College London,<br>Harwood, Hannah; King's College London<br>Greenberg, Karla; King's College London<br>Stevelink, Sharon; King's College London<br>Greenberg , N; King's College London
**Primary Subject Heading**:	Occupational and environmental medicine
Secondary Subject Heading:	Geriatric medicine, Qualitative research
Keywords:	Veteran, Military, Physical health, Geriatric, Occupation

SCHOLARONE™
Manuscripts

The impact of military service on physical health later in life: A qualitative study of geriatric UK veterans and non-veterans.

Victoria Williamson[a*], Hannah Harwood[a], Karla Greenberg[a], Sharon AM Stevelink[a,b+], and Neil Greenberg[a+]

[+] Joint Last Authors

[a] King's Centre for Military Health Research, King's College London, Weston Education Centre, 10 Cutcombe Road, London, SE5 9RJ

b Department of Psychological Medicine, King's College London, 16 DeCrespigny Park, London, postcode

***Correspondence:**

Dr Victoria Williamson

Address: Kings Centre for Military Health Research, King's College London, Weston Education Centre, 10 Cutcombe Road, London, SE5 9RJ, UK

Tel: +44 207 848 5347; Email: Victoria.williamson@kcl.ac.uk

Funding statement: This research was supported by a grant from the Royal British Legion's Aged Veteran's Fund, grant number AVF-TRBL03 (NG). This paper represents independent research part-funded by the National Institute for Health Research (NIHR) Biomedical Research Centre at South London and Maudsley NHS Foundation Trust and King's College

For peer review only - http://bmjopen.bmj.com/site/about/guidelines.xhtml

London (SS). The views expressed are those of the authors and not necessarily those of the NHS, the NIHR or the Department of Health and Social Care.

Competing interests: All authors have completed the ICMJE uniform disclosure form at http://www.icmje.org/coi_disclosure.pdf and declare: authors had financial support from a Royal British Legion grant for the submitted work; no financial relationships with any organisations that might have an interest in the submitted work in the previous 3 years; no other relationships or activities that could appear to have influenced the submitted work.

Data sharing statement: No additional data are available.

Ethics approval: The National Health Service Research Ethics Committee (17/LO/0077).

Acknowledgements: The authors would like to thank Solent NHS Trust and its PPG for the guidance and support they provided throughout this project.

For peer review only

For peer review only - http://bmjopen.bmj.com/site/about/guidelines.xhtml

Abstract

Objectives: Military veterans often experience physical health problems in later life; however, it remains unclear whether these problems are due to military service or are a feature of the ageing process. This study aimed to explore veteran and non-veteran perceptions of the impact of their occupation on their physical wellbeing later in life.

Design: Semi-structured qualitative interviews analysed using thematic analysis.

Setting: Interviews were conducted face-to-face in participants' homes or via telephone.

Participants: 35 veterans (≥65 years); 25 non-veterans (≥65 years) were recruited, as well as a close companion of all participants for triangulation (n=60).

Results: Most veterans reported good physical health later in life which they attributed to the fitness they developed during military service. However, several veterans described challenges in maintaining their desired level of physically activity due to new commitments and limited sports facilities when they left service. Fewer non-veterans had experienced work-related fitness activities or exercise in their civilian jobs. Ongoing physical health difficulties, such as deafness, were perceived to be due to exposure to workplace hazards and appeared more common in veterans compared to non-veterans. Veterans also described greater reluctance than non-veterans to seek medical treatment for physical health difficulties, which could be challenging for close companions who had to provide informal care.

Conclusions: Military service was largely perceived to be beneficial for physical wellbeing; although when occupation-related physical health problems were experienced, many veterans were unwilling to seek treatment. These findings may inform clinicians of the needs of older veterans and highlight potential barriers to care.

Keywords: Veteran, occupation, physical health, geriatric

For peer review only

For peer review only - http://bmjopen.bmj.com/site/about/guidelines.xhtml

Article summary

Strengths and limitations of this study:

- Large-scale qualitative study (n=120) which recruited veterans and non-veterans aged over 65 years and a close companion of each participant.
- Semi-structured, qualitative interviews were conducted face-to-face in participant's homes or via telephone.
- Inclusion of the views of a close companion (spouse, child, close friend) to triangulate the study findings.

For peer review only - http://bmjopen.bmj.com/site/about/guidelines.xhtml

The UK Armed Forces (AF) veteran population is becoming increasingly elderly, with approximately 64% of UK AF veterans over the age of 65 [1,2]. Despite the sizeable and growing elderly UK AF veteran community, relatively little is understood about the impact of military service on the wellbeing of older UK AF veterans. Limited literature, mostly from the US, suggests there may be several health implications from military service [2–4]. However, as over 65s in the general population also commonly suffer from various physical health problems [5,6], whether health issues experienced by older veterans are related to UK military service or are a feature of ageing remains unclear.

The limited available evidence indicates that UK AF veterans may be more likely than the general population to experience physical health conditions that affect their daily functioning. For example, working age UK AF veterans have higher rates of hearing and musculoskeletal problems compared to non-veterans [2]. Nonetheless, military personnel are often fitter and healthier at the time of enlistment than the general population, leading to a phenomenon termed the "healthy warrior effect" [7]. This may explain why a 25% lower mortality risk for older Australian veterans compared with non-veterans has been observed [7]. Moreover, the high-levels of physical activity required in the AF as part of training, sport or deployment activities may offer some protective effects for health conditions later in life, with midlife exercise found to significantly reduce the risk of later high cholesterol and dementia (e.g.[8]). Finally, physical health problems can often have an impact on family members and caregivers. How health problems in working-age individuals can affect their families has been examined in previous studies [9,10], however comparatively little is known about the experiences of caregivers of older ex-military personnel.

A deeper understanding of the perceived long-term impact of AF service on physical health may inform clinical practice, identify whether there is a need to alter the way in which services for this population are provided, and highlight areas for occupational policy change.

For peer review only - http://bmjopen.bmj.com/site/about/guidelines.xhtml

We conducted in-depth, qualitative interviews with older veterans, older non-veterans, and a close companion (i.e. spouse, child, close friend) of each veteran/non-veteran participant. We aimed to explore perceptions of the impact of military vs non-military occupation on physical wellbeing later in life.

Method

Ethical approval for this study was granted by the NHS Camden & King's Cross Research Ethics Committee (17/LO/0077).

Study design

This was a qualitative study using in-depth interviews. The qualitative approach utilised allowed for the exploration of issues that were most salient to participants and the subjective meaning attached to their career and wellbeing. The present study was nested within a larger programme of research [11] examining the impact of military service on physical, psychological and social functioning later in life.

Participants

Four sub-samples of participants were recruited: 1) 25 older veterans (≥65 years, five or more years of regular AF service) not known to have mental health problems; 2) 25 older non-veterans not known to have mental health problems (≥65 years, five or more years of work in a non-military occupation), 3) 10 older veterans with experiences of mental health issues (≥65 years, five or more years of AF service); and 4) a close companion of all participants (≥18 years, n=60). The 25 veterans without mental health diagnoses and 25 non-veterans were age (+/- 5 years) and gender matched. Exclusion criteria included: brain damage or cognitive impairment; inability to speak English; or current self-injurious behaviour/suicidal intent.

Assessments

For peer review only - http://bmjopen.bmj.com/site/about/guidelines.xhtml

Physical health. Veteran and non-veteran physical health and quality of life were measured using the 12-item Short Form Health Survey (SF-12)[12] and the EuroQol (EQ-5D)[13]. Summary scores for the physical health component of the SF-12 were used (maximum possible score of 100), with high scores indicating better health state. The visual analogue scale (VAS) of EQ-5D was used which asks respondents to rate their overall health with 'worst imaginable health state' set at 0 and 'best imaginable health state' set at 100 [13]. The reliability and validity of both measures have been evidenced. These questionnaire assessments were completed by post (n=62) or online (n=58).

Qualitative interview schedule and procedure

The semi-structured interview questions focused on participants perceptions of their career, the impact of their career on their physical health, and their experiences of informal care for physical health problems (see Supplementary file 1). Close companions were asked for their perceptions of the veteran or non-veteran's career and its impact on physical health, as well as their own experiences of caring for the veteran/non-veteran.

The majority of interviews were conducted face-to-face (n=82), with 38 conducted via telephone. All participants gave written (if interview conducted in person) or audio-recorded verbal (if interview conducted via telephone) informed consent for their participation. Interviews were conducted by study researchers who had training and experience in qualitative methods. Interviews lasted for an average of 67 minutes (median = 65 minutes, SD = 0.02, IQR Range = 52:29 – 01:20:56 minutes). All interviews were digitally recorded and transcribed verbatim. Interviews were completed by 120 participants to represent a wide range in occupations and ages to reflect the possible diversity of opinions. Thematic saturation was achieved, and this was determined by the research team when no additional themes were found from the reviewing of successive data [14].

Procedure

For peer review only - http://bmjopen.bmj.com/site/about/guidelines.xhtml

Purposive sampling was used. As previous research has highlighted the relationship between poor mental health and physical health problems [2,15], the aim of the purposive sampling of veterans with self-reported mental health problems was to allow for further exploration of potential links between occupation and health concerns. Veteran and non-veteran participants were initially identified by the clinical care team following attendance at National Health Service (NHS) GP surgeries, walk in centres, community groups or mental health services. The clinical care team identified potentially eligible participants using study inclusion/exclusion criteria. The clinical care team sought permission for patient contact details to be shared with the research team. Given this agreement, participants were contacted by researchers with additional study information. Participants could also self-refer to the study via study advertisements. Companions were recruited by asking veteran/non-veterans if they had a spouse, child or close friend willing to participate with them. Veteran/non-veterans provided their companion's contact details and companions were then contacted by the research team and sent a study information pack.

A total of 86 veteran/non-veteran participants were approached to take part in the study, 60 of which were recruited (69.8% recruitment rate). Eligible individuals who did not participate were largely not contactable or had no close companion to take part. All participants were recruited between March and December 2017. All participants were given a £20 voucher as a thank you for their time.

Data analysis

Quantitative analysis. Data were entered and analysed using the statistical analysis software package SPSS (version 24). Missing data on the EQ-5D VAS and SF-12 were excluded from the analysis (EQ-5D VAS data missing for 3 non-veterans and 1 veteran; SF-12 data missing for 5 non-veterans and 6 veterans). T-tests were used to determine whether

For peer review only - http://bmjopen.bmj.com/site/about/guidelines.xhtml

statistically significant differences between groups existed, with *p* values <0.05 used to indicate statistical significance.

Qualitative analysis. All transcripts were entered into NVivo 11 (QSR International). Data were analysed using thematic analysis using the steps described by Braun and Clarke [16]: reading and re-reading the data, producing codes, searching for and developing early themes, and revising and refining themes. Thematic analysis was utilised as it is a method suitable for larger sample sizes and is analytic strategy used to identify patterns of meaning across the data set as a whole, in keeping with the study's objective of exploring the impact of occupation across the life-course. An inductive analytical approach was used with initial codes and themes proposed by VW. Data collection and analysis took place simultaneously to allow emerging topics of interest to be investigated further in later interviews and to determine whether thematic saturation had been reached[14]. A reflexive journal was kept throughout data collection and analysis by the primary researcher (VW) to recognise the influence of the researcher's prior experiences, thoughts and assumptions and avoid premature or biased interpretations of the data[17]. To ensure reliability, all codes and themes were independently reviewed by authors VW and HH. Disagreements between authors were infrequent and were resolved following discussion and re-examination of the data. Peer debriefing was conducted to further enhance credibility, where discussions were held about the emerging findings and feedback regarding data interpretation and analysis was sought from co-authors SAMS, KG, and NG.

Patient involvement

The study involved patients in shaping the study design and outcome measures as the study materials were piloted with veterans (n=3) and non-veterans (n=2). Patients were also involved in the study recruitment as recommendations for potential recruitment sites were

For peer review only - http://bmjopen.bmj.com/site/about/guidelines.xhtml

sought from Patient Participation Group (PPG) meetings. The findings were disseminated to participants via a study newsletter.

Results

Descriptive information

Participant demographic information can be found in Table 1. Overall, the 35 veterans had between 5-40 years of AF service, with 34.3% serving in the Naval Services, 51.4% serving in the Army, and 14.3% in the Royal Air Force (RAF, see Table 1). Close companions (n=60) were largely spouses (n=46, 76.7%) or close friends (n=8, 13.3%) and had a mean age of 68.4 years (SD 10.6).

Insert Table 1 around here

Differences in scores on the SF-12 and EQ-5D were not statistically significant between veteran, veterans with mental health diagnoses, and non-veteran samples (p>0.05, see Table 1).

Qualitative results

Three key themes emerged from the data regarding experiences and perceived impact of occupation-related exercise, exposure to workplace hazards and the effects on health, and perceptions of and responses to physical health problems. Anonymised participant comments are provided to illustrate the findings in Table 2, and all participants have been given a pseudonym.

Occupation and physical exercise.

For peer review only - http://bmjopen.bmj.com/site/about/guidelines.xhtml

The majority of veterans reported high-levels of physical exercise in the AF, which was not described by non-veterans in civilian roles. Many veterans thought they had been very physically fit due to the military training exercises and sports. This high volume of exercise during their military career was seen as a key reason for veteran good physical health later in life. Many veterans continued to exercise once leaving the AF and keeping physically active was a central part of daily life in retirement. In roles where non-veterans had the opportunity to do some exercise (e.g. manual labour), this activity was considered to have kept them in good health at the time but was often seen as having no long-term benefits.

Nonetheless, several veterans stated they had chronic arthritis and other musculoskeletal health complaints in older age which were thought to be due to the physically demanding activities required in the AF (e.g. parachuting, marching exercises, etc.). These health problems were less commonly described by non-veterans and were not thought to be related to occupation.

On leaving the AF, a minority of veterans experienced difficulties continuing physical activity, either due to the long working hours of their civilian job, being employed in a more sedentary civilian role (e.g. HGV driving, security, etc.), limited access to sports facilities, or due to a military-related physical injury. Veteran difficulties continuing exercise were also discussed by their close companions.

Impact of occupational health and safety.

Participants in both veteran and non-veteran samples reported sustaining physical injuries in the workplace, with the majority of injuries viewed as being due to a lack of protective equipment or poor health and safety regulations (e.g. lack of ear defenders, limited protection available against hazardous substances).

Veterans more frequently reported long-term health difficulties due to a lack of protective equipment compared to non-veterans. Veterans often described experiencing

For peer review only - http://bmjopen.bmj.com/site/about/guidelines.xhtml

hearing loss due to a lack of ear defenders in loud engine rooms or on shooting ranges, as well as skin cancers believed to be caused by having no sun protection during training activities or deployment.

For non-veterans, those in physically demanding jobs often reported strain injuries due to heavy lifting or spending several hours standing. Physically demanding jobs were thought to exacerbate existing physical health conditions (e.g. existing musculoskeletal problems made worse by heavy lifting). Some non-veterans in healthcare roles or jobs requiring foreign travel (e.g. Foreign Office, employment with an international company, etc.) also described that they had contracted serious illnesses (i.e. shingles, dysentery, etc.). Such injuries/illnesses were often felt to have long-lasting effects, with physical pain or disability experienced into retirement.

Impact of occupational hazards on the family. It reportedly became more challenging for close companions to cope with veteran and non-veteran's occupation-related health problems later in life, particularly in cases of veteran/non-veteran hearing loss or debilitating illness. These close companions described feeling lonely as well as frustrated that their plans to exercise or travel as a family in retirement were now not feasible.

The health of close companions or other family members could also reportedly be affected by veteran and non-veteran's exposure to workplace hazards. For example, some close companions discussed being exposed to asbestos on veteran/non-veteran's work clothes. This indirect exposure was thought to cause serious illness, and family members reportedly experienced cancer, infertility, breathing problems and other conditions as a result. When family members became unwell in such circumstances, veterans and non-veterans disclosed experiencing significant guilt and anxiety for their family members' wellbeing, often taking considerable steps to address the perceived harm they had caused (e.g. setting up a charity to help others with similar health problems, becoming their full-time carer, etc.).

For peer review only

Perceptions of and responses to health problems.

Veterans with and without diagnoses of mental health problems described experiencing similar self-reported physical health problems (e.g. arthritis, diabetes, high blood pressure, hearing loss, etc.). Veterans without diagnoses of mental health difficulties more often considered that their physical health conditions related to AF service compared to veterans with mental health diagnoses. Few other differences in terms of physical health problems were described between the two sub-samples.

Reluctance to seek medical treatment for physical health problems both during and following military service was commonly reported in veterans. This reluctance was largely not described by non-veterans. Unwillingness to admit physical health problems and access treatment was reportedly due to a sense of self-sufficiency instilled by the AF and a need to feel and be seen as 'tough.'

Experiences of care and support. Close companions detailed how they play a central role in encouraging veterans to access medical treatment, often accompanying them to appointments and ensuring medication was taken. In cases of serious illness/disability, many close companions of veterans described that veterans were extremely reluctant to accept informal care, such as assistance with getting dressed, daily hygiene, etc., often due to embarrassment or pride, and unwillingness to accept care was less common in non-veterans.

Providing support and care to unwilling veterans could be a distressing and frustrating experience for close companions who often felt their efforts were not appreciated. To cope, close companions positively reframed their experience, for example describing that the veteran would provide them with the same care if needed or seeking support from other family members and friends. Close companions of non-veterans described facing less resistance to their provision of support.

Discussion

For peer review only - http://bmjopen.bmj.com/site/about/guidelines.xhtml

For peer review only

The aim of this study was to explore the perceived impact of military and non-military occupations on physical health later in life. Our findings illustrate the perceived considerable benefits of occupation on wellbeing, particularly for those who served in the AF, such as long-term good physical health as a result of physical exercise. At the same time, these results also highlight the significant long-term implications of workplace practices on wellbeing, including chronic physical health problems due to a lack of protective equipment and beliefs regarding the wider impact of such workplace hazards on an employee's family.

Our findings delineate the positive influences of occupation on health, including the perceived long-lasting benefits of physical exercise in the AF. Physical exercise is associated with lower risk of health problems later in life, including dementia [8] and cardiovascular issues [18]. This may potentially mean AF veterans are at an advantage as opportunities for occupational physical activity were more limited in non-veterans and perceived long-lasting health benefits of occupational exertion in this group were not described. However, as we found no significant differences in physical functioning between veterans and non-veterans on the SF-12 and EQ-5D VAS, this finding must interpreted cautiously. Moreover, some difficulties continuing physical exercise on leaving the AF were experienced due to logistical barriers or musculoskeletal injuries. This is in line with previous research in working age US and UK veterans [15,19,20] and provides insight into why some veterans may be vulnerable to obesity on leaving the AF [21,22]. Interventions to increase physical activity in non-veteran workplaces and additional support for veterans to facilitate exercise on leaving the AF may be beneficial. Nonetheless, it is important to stress that this exercise must be well managed, as veterans also reported experiencing musculoskeletal health complaints which they believed were caused by very strenuous AF physical activity.

Previous research into the impact of military service on physical health has also highlighted that, compared to the general population, working-age veterans are more likely to

For peer review only - http://bmjopen.bmj.com/site/about/guidelines.xhtml

experience hearing difficulties, musculoskeletal problems and arthritis on leaving service [2,23,24]. The findings of this study provide support for these results, offer insight into why such physical health problems potentially occur, for example that hearing loss occurred due to lack of ear defenders, and what the perceived long-term implications can be. As ongoing health problems linked to lack of protective equipment were found in both veteran and non-veteran groups, this may reflect occupational health and safety standards of the time (e.g. 1950-1990's;[25,26]). In veterans, it could also reflect the inability of the Ministry of Defence (MoD) to apply health and safety legislation in an unpredictable conflict zone[27]. Given the adverse impact of workplace hazards, this highlights the importance of effective health and safety procedures (e.g. regular safety inspections, employee training, etc.) and the continued need for the employers to ensure appropriate measures are in place to protect personnel. Participants believed that their family members could also be indirectly exposed to workplace hazards which were thought to contribute to physical health problems (e.g. respiratory difficulties, cancer, etc.). While ascertaining whether family members' health problems were a result of veteran/non-veteran occupational practices or other factors, such as attribution bias, was beyond the scope of this study, these findings are not inconsistent with previous research (e.g.[28]), and suggest a need for continued research regarding the long-term health implications of hazardous work environments. These results also suggest that employers should provide employees family members with accurate information about potential health threats in order to safeguard against potential ill-effects, allay fears and inform them when and how to access medical support.

Another key theme emerged in relation to treatment seeking as veterans appeared to be more reluctant than non-veterans to seek medical treatment for physical health problems or accept informal care - reportedly due to embarrassment or an AF instilled need to be 'tough.' As delays to necessary medical care can adversely impact wellbeing[29], this

For peer review only - http://bmjopen.bmj.com/site/about/guidelines.xhtml

behaviour is concerning. As efforts have been successfully made to encourage help-seeking in those personnel/veterans experiencing mental health difficulties (e.g.[30]), a similar approach for promoting treatment seeking for physical health issues may also be beneficial. Furthermore, close companions often provided support for veterans to access medical treatment as well as informal care. In line with previous research in carers, refusal of care could be a challenging and distressing experience for some [31,32]. Older caregivers who provide care to a reluctant spouse have been found to report long-term marital unhappiness, feelings of entrapment in the role of carer, and distress from witnessing the deterioration in their spouse's condition [33]. Therefore, the families of older veterans may benefit from additional support and guidance related to their role as a caregiver. Further research is needed to ensure the provision of such support is accessible and appropriate.

The current study provided a perspective on an important, yet understudied, group in examining the impact of military service on wellbeing in older UK AF veterans. We recruited a diverse sample of veterans and non-veterans from multiple professions and took steps to ensure the data was robust by triangulation with close companions. At the same time, there are limitations that must be considered. First, this study largely included male veterans/non-veterans, although this is broadly consistent with the gender distribution of the UK AF. Second, this study included only those veterans and non-veterans who agreed to contact by the research team or chose to self-refer in response to study advertisements. This may mean that participants had particularly salient occupation-related issues they wished to discuss. Third, while the key outcome measure of the study was the qualitative interview, a small proportion of participants did not fully complete questionnaire assessments resulting in missing data which must be taken into consideration. Furthermore, the inclusion of non-veterans with diagnoses of mental health problems in future studies would further our understanding of the potential relationship between occupation, physical and mental health.

For peer review only - http://bmjopen.bmj.com/site/about/guidelines.xhtml

Finally, given the qualitative nature of the study, the findings reflect the lived experiences of a sample of veterans, non-veterans, and close companions and large-scale quantitative investigations would be useful in determining the generalizability of the results.

Despite these limitations, this study adds to the very limited research into the long-term physical health implications of military service on older UK veterans in several ways. First, by directly comparing the experiences of veterans and non-veterans, our results suggest that military service may have considerable beneficial effects on physical health later in life due to greater opportunities for exercise. Second, these findings demonstrate that health and safety practices, particularly in the AF, in may lead to physical health problems which can impact long-term functioning. Finally, this research highlights that additional advice and support may be beneficial to veterans and their families to facilitate veteran access to medical treatment which could ultimately improve their wellbeing and reduce familial stress.

For peer review only - http://bmjopen.bmj.com/site/about/guidelines.xhtml

Contributors: VW, SAMS, HH, KG, NG made substantial contributions to the conception of the study and analysis and interpretation of data for the study; drafted the article and revised it critically for important intellectual content; gave final approval of the version to be published and agreed to be accountable for all aspects of the article in ensuring that questions related to the accuracy or integrity of any part of the article are appropriately investigated and resolved. VW and HH contributed to acquisition of the data.

For peer review only

1 NEL Commissioning Support Unit. Developing mental health services for veterans in England engagement report. 2016. https://www.england.nhs.uk/commissioning/wp-content/uploads/sites/12/2016/09/veterans-mh-services-engagement-rep.pdf (accessed 1 Aug 2018).

2 The Royal British Legion. A UK Household Survey of the Ex-Service Community. London: 2014. www.compasspartnership.co.uk

3 Lehavot K, Hoerster KD, Nelson KM, *et al.* Health Indicators for Military, Veteran, and Civilian Women. *Am J Prev Med* 2012;**42**:473–80. doi:10.1016/J.AMEPRE.2012.01.006

4 Bedard K, Deschênes O. The Long-Term Impact of Military Service on Health: Evidence from World War II and Korean War Veterans. *Am Econ Rev* 2006;**96**:176–94. doi:10.1257/000282806776157731

5 Blyth FM, Noguchi N. Chronic musculoskeletal pain and its impact on older people. *Best Pract Res Clin Rheumatol* 2017;**31**:160–8. doi:10.1016/J.BERH.2017.10.004

6 Parker L, Moran GM, Roberts LM, *et al.* The burden of common chronic disease on health-related quality of life in an elderly community-dwelling population in the UK. *Fam Pract* 2014;**31**:557–63. doi:10.1093/fampra/cmu035

7 McLaughlin R, Nielsen L, Waller M. An Evaluation of the Effect of Military Service on Mortality: Quantifying the Healthy Soldier Effect. *Ann Epidemiol* Published Online First: 2008. doi:10.1016/j.annepidem.2008.09.002

8 Ahlskog JE, Geda YE, Graff-Radford NR, *et al.* Physical Exercise as a Preventive or Disease-Modifying Treatment of Dementia and Brain Aging. *Mayo Clin Proc* 2011;**86**:876–84. doi:10.4065/MCP.2011.0252

9 Thandi G, Oram S, Verey A, *et al.* Informal caregiving and intimate relationships: the

For peer review only - http://bmjopen.bmj.com/site/about/guidelines.xhtml

experiences of spouses of UK military personnel. *J R Army Med Corps* 2017;**163**:266–72. doi:10.1136/jramc-2016-000679

10 Beach SR, Schulz R, Yee JL, *et al.* Negative and positive health effects of caring for a disabled spouse: Longitudinal findings from the Caregiver Health Effects Study. *Psychol Aging* 2000;**15**:259–71. doi:10.1037/0882-7974.15.2.259

11 Williamson, V., Harwood, H., Greenberg, K., Stevelink, S., Greenberg N. Veteran and Occupation Impact Evaluation (VOICE) Study. London: 2018. https://www.kcl.ac.uk/kcmhr/publications/reports/files/voice-study-report-2018.pdf (accessed 29 Mar 2019).

12 Ware JE, Kosinski M, Keller SD. A 12 item short form health survey: Construction of scales and preliminary tests of reliability and validity. *Med Care* Published Online First: 1996. doi:10.2307/3766749

13 Hurst NP, Jobanputra P, Hunter M, *et al.* Validity of EUROQOL—a generic health status instrument—in patients with rheumatoid arthritis economic and health outcomes research group. *Br J Rheumatol* 1994.

14 Hennink MM, Kaiser BN, Marconi VC. Code Saturation Versus Meaning Saturation. *Qual Health Res* 2017;**27**:591–608. doi:10.1177/1049732316665344

15 Ashwick R, Murphy D. Exploring the health risks of help-seeking military veterans living in different parts of the UK. *J R Army Med Corps* 2017;**0**:1–7. doi:10.1136/jramc-2017-000822

16 Braun V, Clarke V. Using thematic analysis in psychology. *Qual Res Psychol* 2006;**3**:77–101.

17 Morse JM. Critical Analysis of Strategies for Determining Rigor in Qualitative Inquiry. *Qual Health Res* 2015;**25**:1212–22. doi:10.1177/1049732315588501

18 Lavie CJ, Arena R, Swift DL, *et al.* Exercise and the Cardiovascular System. *Circ Res*

For peer review only

2015;**117**:207–19. doi:10.1161/CIRCRESAHA.117.305205

19 Almond N, Kahwati L, Kinsinger L, *et al.* Prevalence of overweight and obesity among U.S. military veterans. *Mil Med* 2008;**173**:544–9.http://www.ncbi.nlm.nih.gov/pubmed/18595417 (accessed 1 Aug 2018).

20 Johnelle Sparks P, Bollinger M. A Demographic Profile of Obesity in the Adult and Veteran US Populations in 2008. *Popul Res Policy Rev* 2011;**30**:211–33. doi:10.1007/s11113-010-9186-x

21 Ashby M. *How Does Military Experience Influence the Obesity Trend Among Retired Veterans: A Qualitative Grounded Theory Study*. Doctoral Dissertation. The University of the Rockies. 2017.

22 Tarlov E, Zenk SN, Matthews SA, *et al.* Neighborhood Resources to Support Healthy Diets and Physical Activity Among US Military Veterans. *Prev Chronic Dis* 2017;**14**:160590. doi:10.5888/pcd14.160590

23 Okpala NCE. Knowledge and Attitude of Infantry Soldiers to Hearing Conservation. *Mil Med* 2007;**172**:520–2. doi:10.7205/MILMED.172.5.520

24 Williamson V, Diehle J, Dunn R, *et al.* The impact of military service on health and well-being. *Occup Med (Chic Ill)* 2019;**69**:64–70. doi:10.1093/occmed/kqy139

25 Spurgeon A, Harrington JM, Cooper CL. Health and safety problems associated with long working hours: a review of the current position. *Occup Environ Med* 1997;**54**:367–75.http://www.ncbi.nlm.nih.gov/pubmed/9245942 (accessed 1 Aug 2018).

26 Mearns KJ, Flin R. Assessing the state of organizational safety—culture or climate? *Curr Psychol* 1999;**18**:5–17. doi:10.1007/s12144-999-1013-3

27 SHE Health & Safety Software. Time for the MOD to lose its Crown immunity? https://blog.shesoftware.com/time-mod-lose-crown-immunity (accessed 29 Mar 2019).

For peer review only - http://bmjopen.bmj.com/site/about/guidelines.xhtml

28 Rake C, Gilham C, Hatch J, *et al.* Occupational, domestic and environmental mesothelioma risks in the British population: a case–control study. *Br J Cancer* 2009;**100**:1175–83. doi:10.1038/sj.bjc.6604879

29 Prentice JC, Pizer SD. Delayed access to health care and mortality. *Health Serv Res* 2007;**42**:644–62. doi:10.1111/j.1475-6773.2006.00626.x

30 Clark-Hitt R, Smith SW, Broderick JS. Help a Buddy Take a Knee: Creating Persuasive Messages for Military Service Members to Encourage Others to Seek Mental Health Help. *Health Commun* 2012;**27**:429–38. doi:10.1080/10410236.2011.606525

31 Hirst M. Carer distress: A prospective, population-based study. *Soc Sci Med* 2005;**61**:697–708. doi:10.1016/J.SOCSCIMED.2005.01.001

32 Sha, A, Wadoo, O, Latoo J. Psychological distress in carers of people with mental disorders. *Br J Med Pract* 2010;**3**.www.bjmp.org (accessed 29 Mar 2019).

33 Murray, J, Livingston G. A qualitative study of adjustment to caring for an older spouse with psychiatric illness. *Ageing Soc* 1998;**18**:659–71.https://www.cambridge.org/core/journals/ageing-and-society/article/qualitative-study-of-adjustment-to-caring-for-an-older-spouse-with-psychiatric-illness/FD6CAB7E3B9ED18E875502A2220BFA3D (accessed 29 Mar 2019).

For peer review only - http://bmjopen.bmj.com/site/about/guidelines.xhtml

Table 1

Participant demographic information

Demographic		Veterans with MH diagnosis n (%), n=10	Veterans n (%), n=25	Non-veterans n (%), n=25	Close Companions n (%), n=60
Age *M* (SD)		71.8 (6.5)	74.6 (6.9)	75.3 (7.5)	68.4 (10.6)
Gender	Male	7 (70.0)	22 (88.0)	22 (88.0)	9 (15.0)
	Female	3 (30.0)	3 (12.0)	3 (12.0)	51 (85.0)
Ethnicity	White British	10 (100.0)	25 (100.0)	24 (96.0)	59 (98.0)[a]
	Asian/Asian British	n/a	n/a	1 (4.0)	
Service Branch	Naval Services	5 (50.0)	7 (28.0)	n/a	n/a
	Army	4 (40.0)	14 (56.0)		
	RAF	1 (10.0)	4 (16.0)		
Service Length *M* (SD)		20.5 (12.8)	19.6 (8.4)	n/a	n/a
Non-veteran Professions	Doctor/nurse	n/a	n/a	3 (12.0)	n/a
	Managerial/office			6 (24.0)	

For peer review only

	Manual labour			5 (20.0)	
	Small business owner			3 (12.0)	
	Police			1 (4.0)	
	Engineer/scientist			4 (16.0)	
	Civil service			3 (12.0)	
CC Relationship	Spouse	7 (70.0)	16 (64.0)	23 (92.0)	n/a
	Child	1 (10.0)	3 (12.0)	1 (4.0)	
	Close friend	2 (20.0)	5 (20.0)	1 (4.0)	
	Niece/nephew	n/a	1 (4.0)	n/a	
Physical health score *M* (SD)		53.4 (2.4)	54.0 (2.9)	53.6 (2.3)	n/a
Quality of life *M* (SD)		66.4 (16.4)	77.2 (18.1)	70.7 (21.2)	n/a

Note. M= mean. SD= standard deviation. CC= close companion. CC Relationship = the close companion's relationship to the veteran or non-veteran. Veterans with MH diagnoses = veterans with a previous diagnosis of mental health disorders. Service length = number of years in military service. Physical health score = mean score on SF-12 physical health component. Quality of life = mean score on the EQ-5D VAS. Data missing for 3 non-veterans and one veteran (no mental health diagnosis) on the EQ-5D VAS. Data missing for 5 non-veterans and 6 veterans (no mental health diagnoses) on the SF-12. a= this demographic information was missing for one participant. No significant differences between SF-12 scores were found between veterans and veterans with MH diagnoses (t(33)=0.96; p=0.35); veterans without mental disorders and non-veterans (t(48)=-1.20; p=0.24); and non-veterans and veterans with mental health diagnoses (t(33)=0.11; p=0.916). No significant differences

For peer review only - http://bmjopen.bmj.com/site/about/guidelines.xhtml

between EQ5D-VAS scores were found between veterans and veterans with MH diagnoses ($t(32)=1.619$; $p=0.12$); veterans without mental disorders and non-veterans ($t(44)=-1.11$; $p=0.27$); and non-veterans and veterans with mental health diagnoses ($t(30)=0.57$; $p=0.57$).

For peer review only

For peer review only - http://bmjopen.bmj.com/site/about/guidelines.xhtml

Table 2

Themes and sub-themes following thematic analysis

Themes and sub-themes	Findings	Verbatim quotes
Occupation and physical exercise		
High volume of exercise in AF	Veterans reported high-levels of physical exercise during their military career and this was considered a key reason for their good physical health later in life. Few opportunities for exercise were described in non-veterans in civilian roles.	Veteran 1: I've always been pretty healthy, I used to do a lot of sport when I was in the Army…. [Now] I like to go for walks to keep me fit and I [have] a treadmill… So, mentally and physically I'm not too bad for my age.
AF exercise and health problems	The high volume of exercise during military service was thought to have caused chronic health problems in some veterans later in life (e.g. arthritis). These problems were less common in non-veterans and reportedly unrelated to occupation.	Veteran 2: It had an effect on my knees, osteoarthritis… When I joined the Army I did an awful lot of athletics… The Army doesn't do you any good. I mean you've only got to come to a parade here and see the old RSM [Regimental Sergeant Major] banging his feet on the ground and all that sort of thing. And so, my knees are shot.
Difficulties continuing exercise on transition from the AF	Veterans described difficulties in continuing to exercise on transitioning from the military for several reasons, including a lack of sports facilities and more sedentary civilian roles.	Veteran 3: No, I haven't done any real sport since I went outside. There hasn't been the opportunity for it really…The [Sports] Centre here, I need two buses to get to it so... you know, puts you off a bit.
Impact of occupational health and safety		

For peer review only - http://bmjopen.bmj.com/site/about/guidelines.xhtml

Workplace injuries/illnesses	Both veterans and non-veterans described experiencing workplace injuries/illnesses which were often due to poor health and safety regulations. In particular, hearing loss was commonly described by veterans, whereas strain injuries were frequent discussed by non-veterans.	Non-veteran Close Companion 1: Through his plodding around the streets and that…his feet are very bad…They're deformed… He's had waterworks problems, he's got arthritis in both of his shoulders which we think is due to him being out in all weathers policing… he'd be out overnight in all weathers and get drenched and we think that's not helped his arthritis.
Impact of occupational hazards on the family	The health of family members was thought be affected by veteran and non-veteran's exposure to some workplace hazards. Indirect hazard exposure was believed to cause serious illness in family members which often caused veterans/non-veterans to feel guilty and concerned for their wellbeing.	Non-veteran 3: I was on duty… and the nurse in charge…said 'I've heard you're pregnant, great! Don't come any closer, my little boy's got rubella!' And I didn't... I didn't even go into the room, and two weeks later, I had rubella…and that's why my [child] was profoundly deaf.
Perceptions of and responses to health problems		
Reluctance to access care	Reluctance to seek medical treatment for physical health problems both during and following military service was common in veterans. This reluctance was largely not found in non-veterans. Unwillingness to access care was reportedly due to an AF instilled need to feel and be seen as 'tough.'	Veteran 8: It's a man thing! Compounded by the Army. You can't be seen to be a wimp! You are a wimp, but you can't be seen... You've got to keep a stiff upper lip… if you go to anything like an injection, there's no way you flinch! It might hurt, but you don't flinch! No!

For peer review only - http://bmjopen.bmj.com/site/about/guidelines.xhtml

Experiences of care and support	Veteran close companions described that ill/injured veterans were extremely reluctant to accept (in)formal care, often due to embarrassment or pride. Unwillingness to accept care was less common in non-veterans.	Veteran Close Companion 4: He did not enjoy having things done for him, where he couldn't reach his feet or his back to wash properly…So I had to do that for him and he didn't like that. Got very, very upset over that…He thought I was trying to take control of his life. Which I wasn't!... [And] we would have an argument.
Impact of providing care on the family	Providing care to unwilling veterans could be distressing and frustrating for close companions, who often felt their efforts were not appreciated. Close companions of non-veterans described facing less resistance to their provision of support.	Veteran Close Companion 3: [The doctors] found this cancer on him. And then he said 'I'm not having any treatment'… I said 'Look, there's more than you here…You've got [your] grandson…and all [your] friends… You've just got to be a bit considerate'. Anyhow, he had [the treatment].

Note: AF = Armed Forces. All participants have been assigned a pseudonym.

For peer review only - http://bmjopen.bmj.com/site/about/guidelines.xhtml

Supplementary File 1

Topic guide for semi-structured interviews with veterans and non-veterans

Questions
Can you tell me about your employment history?
Probe: Which job(s) would you consider your "main" career?
Probe: How were the early years of your career? What was that experience like?
Probe: What were your main tasks?
Probe: Did you have a say in how you did your work?
Probe: How would you describe your work load?
Probe: What was the most challenging part of this role?
Probe: Were you ever physically injured at work (e.g. shot at, beaten up, threatened to be hurt, exposed to a toxic chemical/substance that could cause you serious harm, etc.)?
Probe: Were you ever involved in a life-threatening automobile accident or another kind of serious accident while at work?
Probe: Did any close colleagues of yours have a traumatic experience while on the job (e.g. assaulted, serious injured, threatened to be hurt)?
Probe: Did you ever do something (either accidentally or on purpose) that led to the serious injury or death of another person while on the job?
Probe: Sometimes people have experiences they don't want to talk about in interviews. Without telling me what it was, did you ever have a difficult or traumatic experience while at work that you haven't mentioned because you don't want to talk about it?
Probe: What were the highlights of this this role?
Probe: What did this role mean to you?
Probe: Why did you do this work?
Probe: What made you go into this type of work?
Are there any skills you feel you've come away with from your job(s)? Why or why not?
Probe: Were there any opportunities open to you as a result of this role that you wouldn't have experienced otherwise (e.g. travel, training, etc.)? Why or why not?
Probe: What has been the most important learning experience in your job? What did it teach you?
How would you describe your physical health over your lifetime?
Probe: How would you describe your current physical health?
Probe: Can you describe any key turning points in your health?

For peer review only

Probe: Have you received a diagnosis for any of your past/present physical health difficulties?

Probe: Do you think any factors had an impact on your physical health? Why or why not?

What impact did your job(s) have on your physical health?

Probe: Have you had any treatment or support for your physical health difficulties? Can you describe your experience of getting this treatment or support?

Generally speaking, how has this type of profession impacted the health of other people?

Can you describe your experience of leaving your profession (e.g. to retire, change roles, etc.)?

Probe: What was retiring/leaving this job like for you?

Probe: Did leaving your profession have an impact on your finances, social or family life?

Probe: How do you feel about your life now that you are retired/have left this job?

Probe: Was there any advice or support that would have been useful to you in making this change?

For all veteran participants:

Have you heard of the military Covenant?

Probe: How would you describe what a 'veteran' is?

Note: Participants were asked open-ended questions and subsequent probing questions depending on their response.

For peer review only

Standards for Reporting Qualitative Research (SRQR)*

http://www.equator-network.org/reporting-guidelines/srqr/

Title and abstract

	Page/line no(s).
**Title** - Concise description of the nature and topic of the study Identifying the study as qualitative or indicating the approach (e.g., ethnography, grounded theory) or data collection methods (e.g., interview, focus group) is recommended	1
**Abstract** - Summary of key elements of the study using the abstract format of the intended publication; typically includes background, purpose, methods, results, and conclusions	3

Introduction

**Problem formulation** - Description and significance of the problem/phenomenon studied; review of relevant theory and empirical work; problem statement	5
**Purpose or research question** - Purpose of the study and specific objectives or questions	6

Methods

**Qualitative approach and research paradigm** - Qualitative approach (e.g., ethnography, grounded theory, case study, phenomenology, narrative research) and guiding theory if appropriate; identifying the research paradigm (e.g., postpositivist, constructivist/ interpretivist) is also recommended; rationale**	8 & 9
**Researcher characteristics and reflexivity** - Researchers' characteristics that may influence the research, including personal attributes, qualifications/experience, relationship with participants, assumptions, and/or presuppositions; potential or actual interaction between researchers' characteristics and the research questions, approach, methods, results, and/or transferability	7 & 9
**Context** - Setting/site and salient contextual factors; rationale**	9
**Sampling strategy** - How and why research participants, documents, or events were selected; criteria for deciding when no further sampling was necessary (e.g., sampling saturation); rationale**	6
**Ethical issues pertaining to human subjects** - Documentation of approval by an appropriate ethics review board and participant consent, or explanation for lack thereof; other confidentiality and data security issues	6
**Data collection methods** - Types of data collected; details of data collection procedures including (as appropriate) start and stop dates of data collection and analysis, iterative process, triangulation of sources/methods, and modification of procedures in response to evolving study findings; rationale**	7 & 8

For peer review only - http://bmjopen.bmj.com/site/about/guidelines.xhtml

For peer review only

**Data collection instruments and technologies** - Description of instruments (e.g., interview guides, questionnaires) and devices (e.g., audio recorders) used for data collection; if/how the instrument(s) changed over the course of the study	7 & 8
**Units of study** - Number and relevant characteristics of participants, documents, or events included in the study; level of participation (could be reported in results)	6
**Data processing** - Methods for processing data prior to and during analysis, including transcription, data entry, data management and security, verification of data integrity, data coding, and anonymization/de-identification of excerpts	8 & 9
**Data analysis** - Process by which inferences, themes, etc., were identified and developed, including the researchers involved in data analysis; usually references a specific paradigm or approach; rationale**	8 & 9
**Techniques to enhance trustworthiness** - Techniques to enhance trustworthiness and credibility of data analysis (e.g., member checking, audit trail, triangulation); rationale**	8 & 9

Results/findings

**Synthesis and interpretation** - Main findings (e.g., interpretations, inferences, and themes); might include development of a theory or model, or integration with prior research or theory	10 - 13
**Links to empirical data** - Evidence (e.g., quotes, field notes, text excerpts, photographs) to substantiate analytic findings	Table 2

Discussion

**Integration with prior work, implications, transferability, and contribution(s) to the field -** Short summary of main findings; explanation of how findings and conclusions connect to, support, elaborate on, or challenge conclusions of earlier scholarship; discussion of scope of application/generalizability; identification of unique contribution(s) to scholarship in a discipline or field	13-17
**Limitations** - Trustworthiness and limitations of findings	16

Other

**Conflicts of interest** - Potential sources of influence or perceived influence on study conduct and conclusions; how these were managed	2
**Funding** - Sources of funding and other support; role of funders in data collection, interpretation, and reporting	1 & 2

*The authors created the SRQR by searching the literature to identify guidelines, reporting standards, and critical appraisal criteria for qualitative research; reviewing the reference lists of retrieved sources; and contacting experts to gain feedback. The SRQR aims to improve the transparency of all aspects of qualitative research by providing clear standards for reporting qualitative research.

For peer review only

For peer review only - http://bmjopen.bmj.com/site/about/guidelines.xhtml

**The rationale should briefly discuss the justification for choosing that theory, approach, method, or technique rather than other options available, the assumptions and limitations implicit in those choices, and how those choices influence study conclusions and transferability. As appropriate, the rationale for several items might be discussed together.

Reference:

O'Brien BC, Harris IB, Beckman TJ, Reed DA, Cook DA. **Standards for reporting qualitative research: a synthesis of recommendations.** *Academic Medicine*, Vol. 89, No. 9 / Sept 2014 DOI: 10.1097/ACM.0000000000000388

For peer review only
